# Muscle pathology of antisynthetase syndrome according to antibody subtypes

**DOI:** 10.1111/bpa.13155

**Published:** 2023-03-07

**Authors:** Jantima Tanboon, Michio Inoue, Shinya Hirakawa, Hisateru Tachimori, Shinichiro Hayashi, Satoru Noguchi, Naoko Okiyama, Manabu Fujimoto, Shigeaki Suzuki, Ichizo Nishino

**Affiliations:** ^1^ Department of Neuromuscular Research, National Institute of Neuroscience National Center of Neurology and Psychiatry (NCNP) Kodaira Tokyo Japan; ^2^ Department of Genome Medicine Development, Medical Genome Center National Center of Neurology and Psychiatry (NCNP) Kodaira Tokyo Japan; ^3^ Department of Clinical Data Science, Clinical Research & Education Promotion Division National Center of Neurology and Psychiatry (NCNP) Kodaira Tokyo Japan; ^4^ Department of Dermatology, Graduate School of Medical and Dental Sciences Tokyo Medical and Dental University Tokyo Japan; ^5^ Department of Dermatology, Faculty of Medicine University of Tsukuba Tsukuba Ibaraki Japan; ^6^ Department of Dermatology, Graduate School of Medicine Osaka University Suita Osaka Japan; ^7^ Department of Neurology Keio University School of Medicine Tokyo Japan; ^8^ Department of Clinical Genome Analysis, Medical Genome Center National Center of Neurology and Psychiatry (NCNP) Kodaira Tokyo Japan; ^9^ Present address: Department of Pathology, Faculty of Medicine, Siriraj Hospital Mahidol University Bangkok Thailand; ^10^ Present address: Department of Neurology Washington University School of Medicine Saint Louis Missouri USA

**Keywords:** antisynthetase syndrome, HLA‐DR, inflammatory myopathy, MHC class II, muscle biopsy, muscle pathology

## Abstract

Identification of antisynthetase syndrome (ASS) could be challenging due to inaccessibility and technical difficulty of the serology test for the less common non‐Jo‐1 antibodies. This study aimed to describe ASS antibody‐specific myopathology and evaluate the diagnostic utility of myofiber HLA‐DR expression. We reviewed 212 ASS muscle biopsies and compared myopathologic features among subtypes. Additionally, we compared their HLA‐DR staining pattern with 602 non‐ASS myositis and 140 genetically confirmed myopathies known to have an inflammatory component. We used *t*‐test and Fisher's exact for comparisons and used sensitivity, specificity, positive and negative predictive values to assess the utility of HLA‐DR expression for ASS diagnosis. RNAseq performed from a subset of myositis cases and histologically normal muscle biopsies was used to evaluate interferon (IFN)‐signaling pathway‐related genes. Anti‐OJ ASS showed prominent myopathology with higher scores in muscle fiber (4.6 ± 2.0 vs. 2.8 ± 1.8, *p* = 0.001) and inflammatory domains (6.8 ± 3.2 vs. 4.5 ± 2.9, *p*  = 0.006) than non‐OJ ASS. HLA‐DR expression and IFN‐γ‐related genes upregulation were prominent in ASS and inclusion body myositis (IBM). When dermatomyositis and IBM were excluded, HLA‐DR expression was 95.4% specific and 61.2% sensitive for ASS with a positive predictive value of 85.9% and a negative predictive value of 84.2%; perifascicular HLA‐DR pattern is common in anti‐Jo‐1 ASS than non‐Jo‐1 ASS (63.1% vs. 5.1%, *p* < 0.0001). In the appropriate clinicopathological context, myofiber HLA‐DR expression help support ASS diagnosis. The presence of HLA‐DR expression suggests involvement of IFN‐γ in the pathogenesis of ASS, though the detailed mechanisms have yet to be elucidated.

## Graphical abstract

Muscle pathology in anti‐OJ ASS is more prominent than the other ASS subtypes.

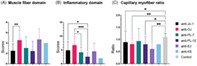

## 1 INTRODUCTION

Antisynthetase syndrome (ASS) is now recognized as one of the major subtypes of autoimmune myositis [1, 2]. ASS has been defined by the presence of anti‐aminoacyl transfer RNA synthetase (antisynthetase, anti‐ARS) autoantibodies with various combinations of clinical features (i.e., myositis, interstitial lung disease (ILD), mechanic's hands, arthritis/arthralgia, fever, and Raynaud phenomenon) [3, 4]. Anti‐Jo‐1 ASS is the prototype while clinically‐associated non‐Jo‐1 ASS (i.e., ASS associated with anti‐PL‐7, anti‐PL‐12, anti‐EJ, anti‐OJ, anti‐KS, anti‐Ha, and anti‐Zo antibodies) are not well‐described. This is likely due to the much higher prevalence of anti‐Jo‐1 antibody; it was present in 593 (72%) of 828 ASS patients in a collaborative American and European cohort [5]. Anti‐Jo‐1 was also the most common antibody present in 306 (18.7%) of 1637 autoimmune myositis in European patients; non‐Jo‐1 anti‐ARS antibodies (excluding anti‐Ha) were collectively identified in 57 (3.5%) patients in the cohort [6].

Using Bohan and Peter classification, ASS was likely categorized as polymyositis (PM) and dermatomyositis (DM) [7]. Notably, a clustering analysis of autoimmune myositis based on clinico‐serological features showed that 95% and 5% of the “ASS‐corresponding cluster” were historically diagnosed with PM and DM, respectively [1]. By this approach, the diagnosis of PM is obsolete. DM features including DM skin lesions (i.e., Gottron signs/papules and/or heliotrope rashes) and perifascicular atrophy (PFA) were reported in 15%–28% [8, 9] and 17.0%–44.4% [10, 11] of ASS patients at the time of diagnosis, respectively. Furthermore, ASS features including mechanic's hands and ILD, were reported in 42.7% and 76.5% of anti‐MDA5 DM, respectively [12]. Pathological features originally described in ASS including perifascicular and perimysial pathology were also reported in DM, especially anti‐Mi‐2 DM [11, 13, 14]. Thus, incomplete serology test and possible technical challenges could misclassify ASS as DM and vice versa [15, 16]. This study aimed to describe myopathological features of ASS to help identify patients in such situations. We also chose to explore myofiber human leukocyte antigen (HLA)‐DR expression as a potential diagnostic tool for ASS because (i) HLA‐DR has been reported mainly in inclusion body myositis (IBM) and ASS and less commonly present in other entities [10, 17, 18] and (ii) other pathological features previously described in ASS can be present in other entities, especially DM [11, 14].

## 2 METHODS

### 2.1 Patients

This study was an expansion of ASS cohort from previous studies [19, 20, 21] including 212 muscle biopsies from serologically confirmed ASS patients evaluated at NCNP, a nation‐wide referral center for muscle disease, between January 2009 and September 2019 (anti‐Jo‐1 = 65, anti‐OJ = 20, anti‐PL‐7 = 20, anti‐PL‐12 = 11, anti‐EJ = 10, anti‐KS = 1, and anti‐ARS positive, not otherwise specified (anti‐ARS_NOS = 85). For comparison on HLA‐DR expression, we included 742 muscle biopsies from 188 DM (anti‐TIF1‐γ = 65, anti‐Mi‐2 = 30, anti‐MDA5 = 22, anti‐NXP‐2 = 60, anti‐SAE = 5, and seronegative DM = 6), 313 immune‐mediated necrotizing myopathy (IMNM, anti‐SRP = 188 and anti‐HMGCR = 125), and 140 myopathies possibly contain inflammatory features [22] categorized as “possible myositis mimics” (P‐MM: dysferlinopathy = 50, sarcoglycanopathy = 15, laminopathy = 16, anoctamin‐5 (ANO5) myopathy = 3, fukutin‐related protein (FKRP) myopathy = 9, and facioscapulohumeral muscular dystrophy (FSHD) = 47) and recently diagnosed IBM (between January 2019 and September 2019, *n* = 101). Patients were classified as adult if they were ≥18 years old.

### 2.2 Serological information and inclusion criteria for autoimmune myositis

Anti‐ARS antibodies and DM‐specific antibodies (DMSA: anti‐TIF1‐γ, anti‐Mi‐2, anti‐MDA5, anti‐NXP‐2, and anti‐SAE) were identified using previously described methods [11]. ASS muscle biopsies were classified according to anti‐ARS subtypes. Patients who tested positive on an ELISA using recombinant ARS antigens (Jo‐1, PL‐7, PL‐12, EJ, and KS) but had no further test(s) for specific antibody subtype were categorized as “anti‐ARS_NOS.” All patients with anti‐OJ antibody were identified by RNA immunoprecipitation; seven cases were selectively tested in patients with clinicopathological impressions of autoimmune myositis who were negative for the other myositis‐specific antibodies. DM muscle biopsies were identified using sarcoplasmic immunoexpression for myxovirus resistance protein A (MxA) with one concurrent DMSA seropositivity or MxA‐positive but negative for all DMSA (seronegative DM) [11]. IMNM muscle biopsies included all patients with either anti‐SRP or anti‐HMGCR seropositive [22]. In Japan, anti‐SRP antibodies were detected by RNA immunoprecipitation and/or ELISA, while anti‐HMGCR antibodies were detected by protein immunoprecipitation and/or ELISA [23, 24]. Anti‐SRP antibodies were also recognized by commercialized immunoblot. We identified IBM by Llyod et al. criteria [25] and used the patchy/dot‐like pattern of p62 as a surrogate marker for rimmed vacuoles.

### 2.3 Possible myositis mimics (P‐MM)

Targeted resequencing gene panels was used to identify dysferlinopathy, sarcoglycanopathy, laminopathy, ANO5 myopathy, and FKRP myopathy [26]. All dysferlinopathy and sarcoglycanopathy muscle biopsies showed decreased or absence of the corresponding proteins on immunohistochemistry and western blotting. FSHD type 1 was diagnosed based on D4Z4 repeat contractions with the 4qA haplotype, and FSHD type 2 was diagnosed by Sanger sequencing [27, 28].

### 2.4 Pathological evaluation

We routinely performed a battery of histochemical and immunohistochemical staining procedures for diagnostic pathologic evaluation, including hematoxylin and eosin, modified Gömöri trichrome, acid phosphatase, alkaline phosphatase (ALP), cytochrome c oxidase (COX), MxA, HLA‐ABC, HLA‐DR, and membrane attack complex (C5b‐9); all of which were re‐evaluated for this study. For ASS, the following immunohistochemical stains also routinely performed for diagnosis, were evaluated: neonatal myosin heavy chain, utrophin, CD3, CD8, CD20, and CD68. We also evaluated p62 in IBM. The clones and sources of antibodies were described in eTable S1.

**FIGURE 1:**
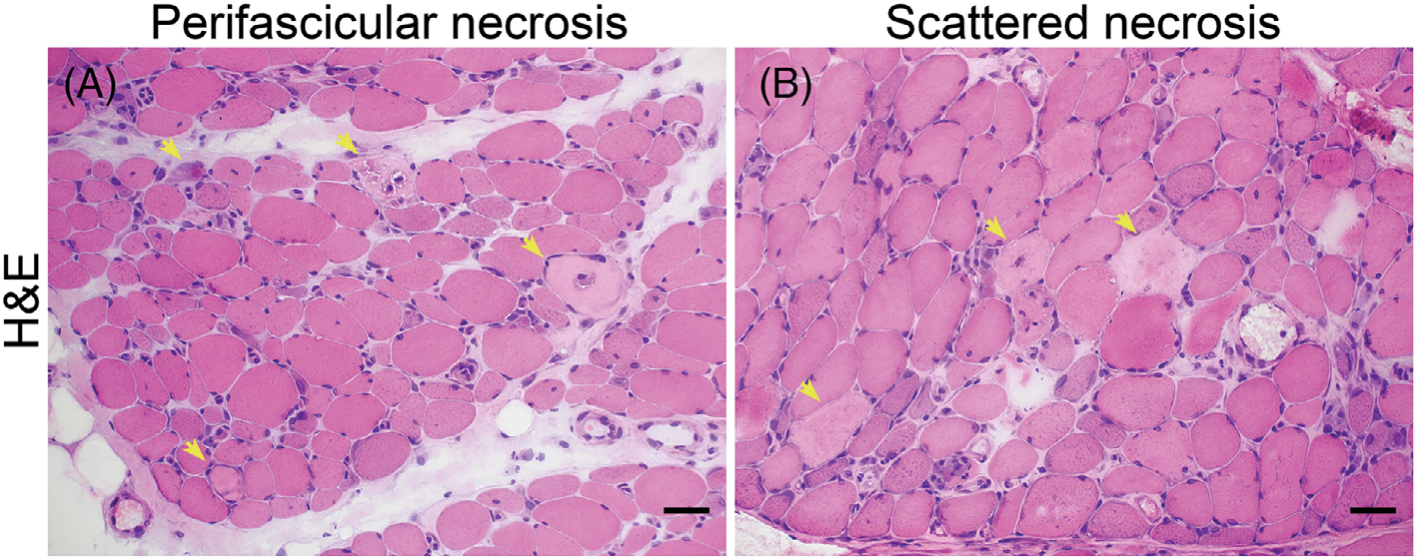
Perifascicular necrosis (PFN): (A) PFN is recognized when two‐third of necrotic fibers present in the two outermost layers at the periphery of the fascicles (perifascicular areas). (B) Scattered necrosis: no specific localization of necrotic fibers. Necrotic fibers are highlighted (yellow arrows) (Bar = 50 μm).

**FIGURE 2:**
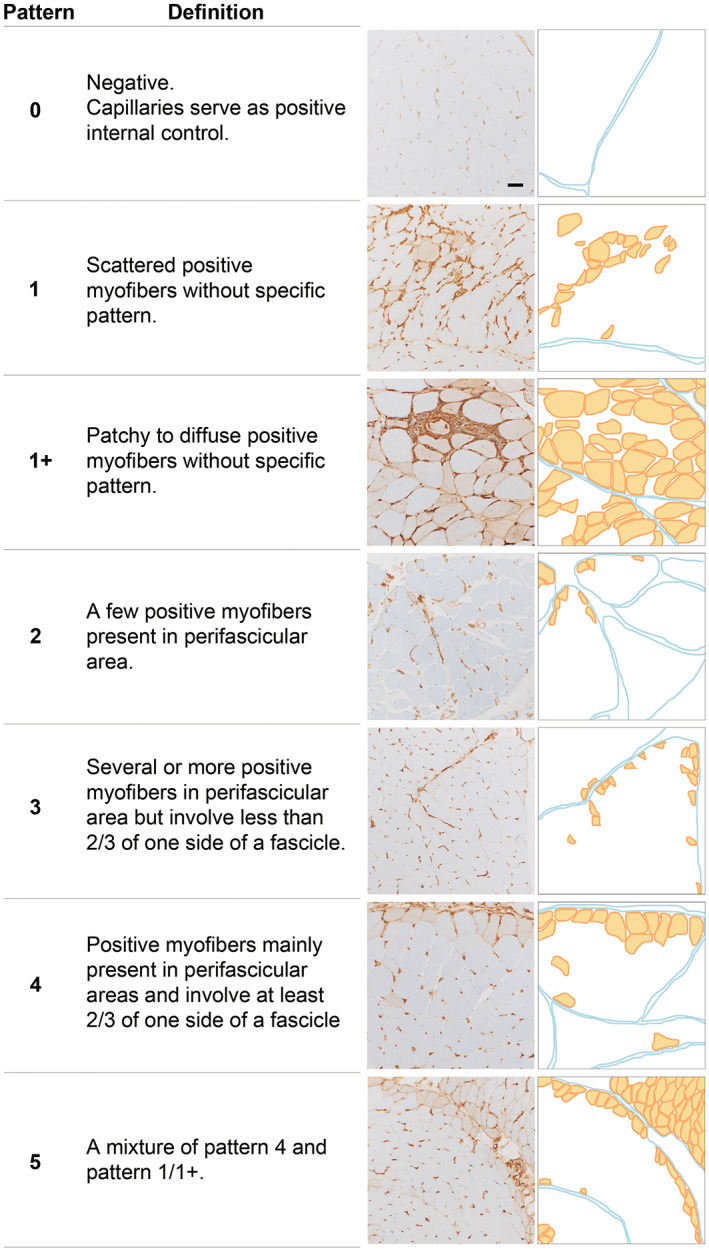
HLA‐DR staining patterns in this study **(**Bar = 50 μm).

JT blindly evaluated muscle biopsies without antibody information. IN confirmed the pathologic findings. The pathology domains (muscle fiber, inflammatory, vascular, and connective tissue) inspired by a pathology scoring system for juvenile DM were evaluated [11] (eTable S2). Because the limited number of juvenile ASS, we excluded them from the capillary: myofiber ratio (CFR) comparison. We categorized ASS into four pathological patterns: (i) normal/non‐specific change, (ii) necrotizing myopathy with perifascicular necrosis (PFN) (Figure 1) [29], (iii) necrotizing myopathy without PFN, and (iv) others (including but not limited to type 2 fiber atrophy, PFA, rimmed vacuoles, nemaline body, cytoplasmic body, and neurogenic change without other distinct pathology). HLA‐DR expression was classified into seven patterns (Figure 2): 0 = negative, 1 = scattered without a specific pattern, 1+ = patchy to diffusely positive without a specific pattern, 2 = a few positive fibers in the perifascicular area, 3 = several or more positive fibers in the perifascicular area, 4 = positive fibers mainly located in the perifascicular area and involving at least 2/3 of one side of a fascicle, 5 = a mixture of pattern 4 and pattern 1/1+. We grouped HLA‐DR categories 3 + 4 + 5 as “perifascicular pattern” since we speculated that these patterns are in continuum. We did not include pattern 2 in “perifascicular pattern” because we suspected that such findings could be randomly present. Ultrastructural evaluation for tubuloreticular inclusions (TRI) was performed in 35 available specimens. The biopsies without TRI observed in 15 randomly examined capillaries were classified as “negative for TRI.”

### 2.5 Statistical analysis

Anti‐KS and anti‐ARS_NOS ASS were included for total ASS features evaluation but were excluded from subtype comparison because the limited number of the former and the possible mixed population of the latter. We used Brown‐Forsythe ANOVA followed by Dunnett T3 test for multiple comparisons of the continuous variables. Welch *t* test and Fisher's exact test were used to compare the continuous and categorical variables, respectively. We compared the percentages of HLA‐DR expression and the expression patterns between ASS and non‐ASS diseases. The sensitivity, specificity, positive predictive value (PPV), and negative predictive value (NPV) of all HLA‐DR expression patterns, including perifascicular (pattern 3 + 4 + 5). The clinical and pathological differences between anti‐Jo‐1 ASS and anti‐OJ ASS with and without perifascicular HLA‐DR expression were compared. Statistical significance was defined by *p* values <0.05. Statistical analyses were conducted using GraphPad Prism version 9.1.0 for MAC (GraphPad).

### 2.6 RNA sequencing

We performed RNA sequencing analysis using frozen muscle biopsy samples from 140 autoimmune myositis patients, including 26 patients with ASS (anti‐Jo‐1 = 13 and anti‐OJ = 13), 81 with DM (anti‐ TIF1‐γ = 18, anti‐Mi‐2 = 14, anti‐NXP‐2 = 32, and anti‐MDA5 = 17), 24 with IMNM (anti‐SRP = 12 and anti‐HMGCR = 12), and 9 with IBM. Twelve histologically normal muscle biopsies were analyzed. In brief, RNA was prepared using TRIzol and Maxwell® RSC Simply RNA Kit (Promega, Madison, WI) and sequenced using the Illumina Hiseq 4000 (Illumina) or MGISEQ‐2000 (Beijing Genomics Institution). Salmon version 0.13.1 was used to align reads and quantify gene abundance [30]. Differential gene expression analysis was performed using DESeq2, version 1.34.0 [31]. Adjusted *p* values ≤0.05 were considered to indicate statistical significance. Differentially expressed genes were ranked according to their degree of significance based on their adjusted *p* value.

### 2.7 Interferon genes and pathway

The lists of interferon (IFN) signaling pathway genes were collected from Reactome biological pathways (reactome.org). R version 4.1.3 [32], RStudio version 2022.02.01 [33], and the ComplexHeatmap package version 2.10.0 [34] were used to create a differential gene expression heatmap.

### 2.8 Standard protocol approvals, registrations, and patient consents

The institutional review board of the NCNP approved this study. All materials used in this study were obtained for diagnostic purposes and were permitted for research use with written informed consent.

## 3 RESULTS

### 3.1 Clinical features

The clinical features were summarized in Table 1. Anti‐OJ ASS were older, had higher creatine kinase (CK) levels, and shorter disease duration before muscle biopsy (non‐OJ ASS 58.3 ± 15.9 years*, p* = 0.03; 2930.0 ± 3476.3 U/L, *p* = 0.03; and 29.7 ± 65.1 months, *p* = 0.03, respectively). Anti‐PL‐12 ASS had lower CK levels. (non‐PL‐12 ASS 3651.9 ± 4130.0, *p* = 0.003). Fever was less common in anti‐Jo‐1 (non‐Jo‐1 ASS 37.1%, *p* = 0.02). The prevalence of muscle weakness, mechanic's hands, Raynaud phenomenon, joint involvement, ILD, and malignancy did not differ among ASS subtypes. DM skin lesions (i.e., Gottron sign/papule and/or heliotrope rash), were observed in 28.3% of ASS patients and were more common in anti‐PL‐7 ASS (non‐PL‐7 ASS 21.5%, *p* = 0.05).

**TABLE 1:** Clinical features of antisynthetase syndrome in this study.

	Anti‐ARS antibody							
	All ARS	Jo‐1	OJ	PL‐7	PL‐12	EJ	KS	ARS_NOS
	(*n* = 212)	(*n* = 65)	(*n* = 20)	(*n* = 20)	(*n* = 11)	(*n* = 10)	(*n* = 1)	(*n* = 85)
Age at biopsy (years)	59.6 ± 15.9	59.1 ± 16.5	66.2 ± 13.3*	55.0 ± 14.8	60.5 ± 12.4	62.4 ± 11.5	13.0	59.7 ± 16.2
Adult (≥18 years old)	208 (98.1)	64 (98.5)	20 (100.0)	19 (95.0)	11(100.0)	10 (100.0)	0	84 (98.8)
Woman	128 (60.4)	40 (61.5)	9 (45.0)	11 (55.0)	5 (45.5)	6 (60.0)	1 (100.0)	56 (65.9)
CK (U/L)	3100.0 ± 3792.8	2742.3 ± 3025.8	6105.6 ± 5724.3*	4484.7 ± 4485.5	1091.6 ± 2146.2*	1866.2 ± 1642.4	14,900	2606.8 ± 3322.7
Disease duration before biopsy (months)	24.8 ± 55.3^a^	30.0 ± 70.6^a^	10.2 ± 26.3*	17.4 ± 33.0	38.2 ± 72.5	47.2 ± 773.7	5	21.9 ± 46.0
Muscle weakness	197 (92.9)	61 (93.8)	20 (100.0)	19 (95.0)	10 (90.9)	10 (100.0)	1	76 (89.4)
Immunotherapy at the time of biopsy	53 (25.0)	12 (18.5)	6 (30.0)	5 (25.0)	2 (18.2)	1 (10.0)	0	27 (31.8)
DM skin lesion^b^	60 (28.3)	13 (20.0)	4 (20.0)	9 (45.0)*	3 (27.3)	2 (20.0)	1	28 (32.9)
Mechanic's hands	57 (26.9)	15 (23.1)	2 (10.0)	4 (20.0)	2 (18.2)	2 (20.0)	0	32 (37.6)
Raynaud's phenomenon	10 (4.7)	2 (3.1)	0	0	0	1 (10)	0	7 (8.2)
Joint involvement	47 (22.2)	21 (32.3)	4 (20.0)	4 (20.0)	3 (27.3)	2 (20.0)	0	13 (15.3)
Interstitial lung disease	156 (73.6)	43 (66.2)	17 (85.0)	18 (90.0)	6 (54.5)	10 (100.0)	0	61(71.8)
Fever	57 (26.9)	11 (16.9)*	7 (35.0)	8 (40.0)	4 (36.4)	3 (30.0)	1	23 (27.1)
Malignancy	17 (8.0)	6 (9.2)	3 (15.0)	0	1 (9.1)	0	0	7 (8.2)

*Note*: Continuous data is shown as mean and ± standard deviation while categorial data is reported as number and (percentage).

Abbreviations: ARS, anti‐tRNA synthetase; CK, creatine kinase; DM, dermatomyositis; NOS, not otherwise specified.

^a^ Information was not available in one case.

^b^ DM skin lesions as defined by the 239th ENMC workshop include Gottron sign/papule and/or heliotrope rash.

* *p* < 0.05 compared to the other antibody subtypes.

### 3.2 Ultrastructural study

TRIs were observed in 11 (31.4%) of ASS muscle biopsies (anti‐Jo‐1, 2/10; anti‐OJ, 2/5; anti‐PL‐7, 1/3; anti‐PL‐12, 4/5; anti‐EJ, 0/3; and anti‐ARS_NOS, 2/9).

### 3.3 Anti‐OJ ASS pathology was prominent in all four domains

**FIGURE 3:**
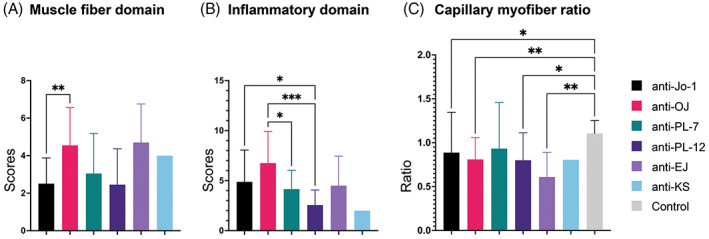
Pathology domains among antisynthetase antibody subtypes. (A) Muscle fiber domain: Anti‐OJ had a distinctively higher muscle fiber domain score than anti‐Jo‐1. (B) Inflammatory domain: Anti‐OJ presented the highest inflammatory domain score which was distinctively higher than anti‐PL‐7 and anti‐PL‐12. Anti‐Jo‐1 showed distinctively higher score than anti‐PL‐12. (C) Adult capillary:myofiber ratio: The ratio in all antisynthetase antibody subtypes, except for anti‐PL‐7, was distinctively lower than controls. The ratio was not distinctively different among antibody subtypes. Bar = mean ± SD, analysis of variance with Dunnett T3 multiple comparison: *p* value 0.0332 (*), 0.0021 (**), 0.0002 (***), <0.0001 (****).

**FIGURE 4:**
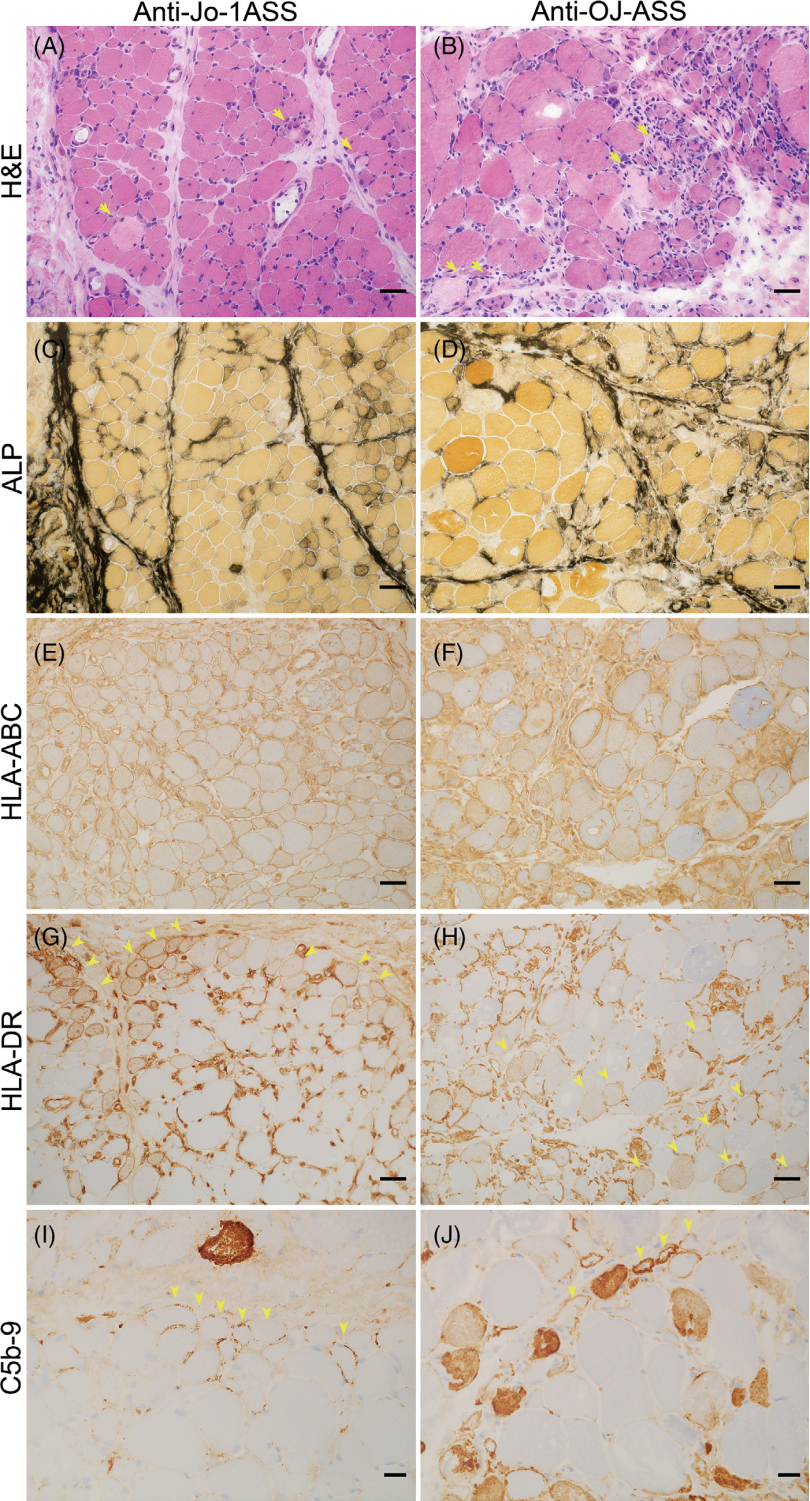
Histologic patterns in anti‐Jo‐1 and anti‐OJ antisynthetase syndrome. Representative figures for anti‐Jo‐1 ASS (A, C, E, G, I) and anti‐OJ ASS (B, D, F, H, J): Both anti‐Jo‐1 and anti‐OJ showed perifascicular necrosis on H&E (yellow arrows indicate necrotic fibers, A and B), but more prominent inflammatory cell infiltration was observed in anti‐OJ ASS (B). Both anti‐Jo‐1 and anti‐OJ ASS showed increased perimysium alkaline phosphatase activity (C and D). HLA‐ABC was diffusely expressed (E and F). Anti‐Jo‐1 ASS was commonly associated with perifascicular HLA‐DR expression (G). In this case, anti‐OJ ASS showed scattered HLA‐DR positivity (H). Both subtypes showed sarcolemmal C5b‐9 expression in perifascicular areas (yellow arrowheads indicate immunohistochemical positive fibers, I and J). A–C, E–G bars = 50 μm; D and H bars = 20 μm; H&E, hematoxylin and eosin; ALP, alkaline phosphatase; HLA‐ABC, human leukocyte antigen‐ABC; HLA‐DR, human leukocyte antigen‐DR; C5b‐9: membrane attack complex.

Anti‐OJ ASS had higher muscle fiber domain, necrotic fiber, regenerating fiber, and PFA scores (4.6 ± 2.0 vs. non‐OJ ASS 2.8 ± 1.8, *p* = 0.001; 1.7 ± 0.6 vs. 1.0 ± 0.7, *p* < 0.0001; 0.9 ± 0.4 vs. 0.6 ± 0.5, *p* = 0.008; and 0.7 ± 0.9 vs. 0.2 ± 0.6, *p* = 0.04, respectively) (eTables S3 and S4, Figures 3A and 4).

Anti‐Jo‐1 ASS had lower muscle fiber domain, necrotic fiber, atrophic fiber, and PFA scores (2.5 ± 1.4 vs. non‐Jo‐1 ASS 3.7 ± 2.2, *p* = 0.0003; 0.9 ± 0.7 vs.1.3 ± 0.7, *p* = 0.002; 0.6 ± 0.7 vs. 0.9 ± 0.9, *p* = 0.03; 0.1 ± 0.4 vs. 0.5 ± 0.8, *p* = 0.0004, respectively). Anti‐OJ ASS was associated with a higher inflammatory score, endomysial and perimysial CD68 infiltration scores, perivascular inflammatory cell infiltration score, and a higher prevalence of perivascular inflammatory cell infiltration and vasculitis (6.8 ± 3.2 vs. non‐OJ ASS 4.5 ± 2.9, *p* = 0.006; 1.9 ± 0.4 vs.1.6 ± 0.6, *p* = 0.02; 1.6 ± 0.7 vs. 1.0 ± 0.8, *p* = 0.0007; 0.8 ± 0.4 vs. 0.4 ± 0.5, *p* = 0.002; 75.0% vs. 36.5%, *p* = 0.002; 45.0% vs. 11.2%, *p* = 0.0009, respectively) (Figure 3B, eTables S3, S5 and S6). Anti‐PL‐12 ASS was associated with lower inflammatory score (2.6 ± 1.5 vs. non‐PL‐12 ASS 5.0 ± 3.1, *p* = 0.0008).

For the vascular domain, the CFR in adult patients of all ASS subtypes except anti‐PL‐7 ASS was lower than that of the control specimen (control CFR 1.1 ± 0.1) (Figure 3C and Figure S1). The adult CFR were not distinctively different among ASS subtypes (eTable S3). Anti‐EJ was associated with high muscle domain score (4.7 ± 2.1 vs. non‐EJ ASS 3.0 ± 1.8, *p* = 0.03) but the inflammatory score was not distinctively different from the other subtypes. For the connective tissue domain, anti‐OJ and anti‐EJ ASS showed more frequent increments in perimysial ALP activity (70.0% vs. non‐OJ ASS 42.1%, *p* = 0.03, 90.0% vs. non‐EJ ASS 42.7%, *p* = 0.006). Endomysial fibrosis was more common in the anti‐EJ ASS group (60.0% vs. non‐EJ ASS 12.0%, *p* = 0.001). Perimysial ALP activity and endomysial fibrosis were less common in anti‐Jo‐1 ASS (33.8% vs. non‐Jo‐1 ASS 59.7%, *p* = 0.004 and 7.7% vs. 24.2%, *p* = 0.01, respectively). The disease duration in patients with and without endomysial fibrosis was not distinctively different (36.8 ± 70.0 vs. 22.5 ± 52.0 months, *p* = 0.27). Notably, patients with endomysial fibrosis had higher muscle domain (4.6 ± 1.6 vs. 2.6 ± 1.7, *p* < 0.0001) and inflammatory domain scores (12.6 ± 5.0 vs. 7.1 ± 5.0, *p* < 0.0001). Perimysial connective tissue fragmentation did not differ among ASS subtypes (eTable S3).

### 3.4 Necrotizing myopathy was the most common myopathological pattern in ASS

**FIGURE 5:**
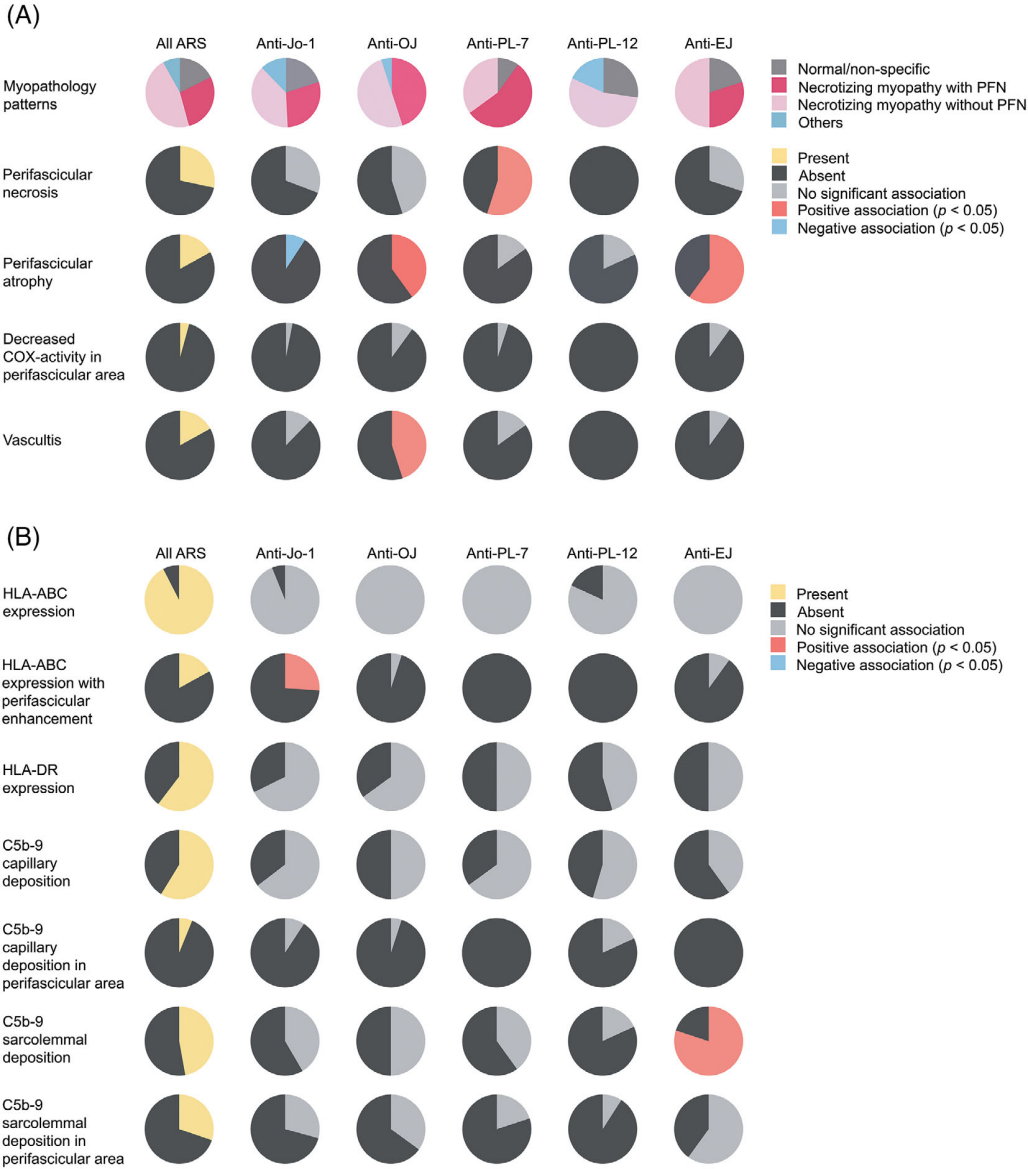
Myopathological (A) and immunohistochemical features (B) in antisynthetase syndrome.

The most common myopathological pattern in ASS patients was necrotizing myopathy without PFN (46.2%) of which the prevalence did not differ substantially among the ASS subtypes. PFN was present in 28.3% of the overall ASS cases and was more common in anti‐PL‐7 ASS (55.0% vs. non‐PL‐7 30.8%, *p* = 0.04). PFN was not present in the anti‐PL‐12 ASS group (0% vs. non‐PL‐12 37.9%, *p* = 0.008). PFA was present in 17% of all ASS; it was common in anti‐OJ (40.0% vs. non‐OJ 16.8%, *p* = 0.03) and anti‐EJ ASS (60.0% vs. non‐EJ 17.1%, *p* = 0.005). Decreased COX activity in perifascicular fibers. CD8 or CD68/acid phosphatase‐positive cell infiltration into non‐necrotic fibers, and CD20‐positive cell aggregation were not common in ASS and were not distinctively different among ASS subtypes (Figure 5A, eTable S6).

### 3.5 Perifascicular HLA‐ABC enhancement and HLA‐DR localization was common in anti‐Jo‐1 ASS

**FIGURE 6:**
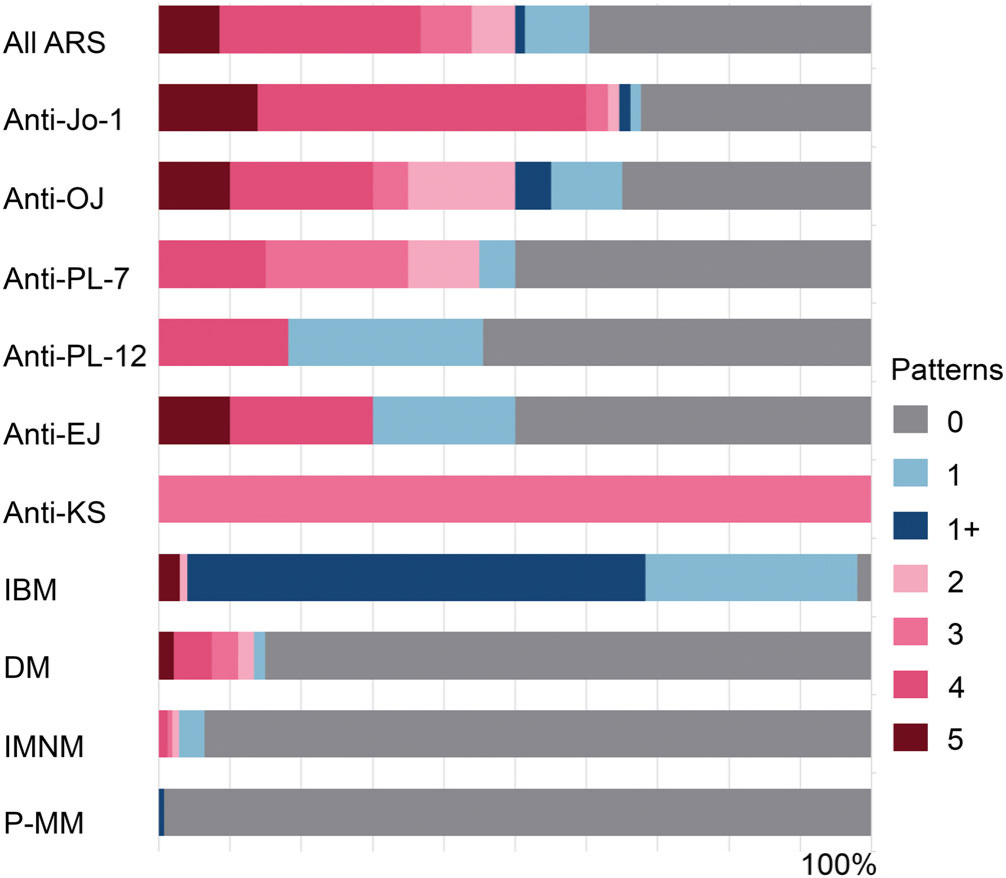
HLA‐DR staining pattern in different entities: HLA‐DR expression in perifascicular area (pattern 3 + 4 + 5) was more common in ASS than the other idiopathic inflammatory myopathies and potentially myositis mimics. The pattern was more common in anti‐Jo‐1 ASS than the other antisynthetase syndrome antibodies. ARS, antisynthetase syndrome (all subtypes); DM, dermatomyositis; IBM, inclusion body myositis; IMNM, immune‐mediated necrotizing myopathy; P‐MM, possible myositis mimics.

HLA‐ABC expression was present in 92.5% of ASS muscle biopsies, and HLA‐ABC expression with perifascicular enhancement was more common in anti‐Jo‐1 ASS (26.2% vs. non‐Jo‐1 3.2%, *p* = 0.0003) (Figure 5B, eTable S7 and eFigure S2). Among 16 HLA‐ABC‐negative ASS biopsies, only one biopsy contained necrotic and regenerating fibers. HLA‐DR expression was observed in 60.4% of ASS patients. All but one HLA‐DR‐positive biopsy showed HLA‐ABC expression. The most common HLA‐DR expression pattern was perifascicular localization that involved at least 2/3 of a fascicle (pattern 4) (28.3%); the pattern was distinctively common in anti‐Jo1 ASS (46.2% vs. non‐Jo‐1 17.7%, *p* = 0.0007) (Figure 6, eTable S8). Pattern 4 HLA‐DR expression was observed in 40.7% (24/59) of ASS muscle biopsies showing necrotizing myopathy with PFN, 29.9% (29/97) of necrotizing myopathy without PFN, 16.2% (6/37) of normal/nonspecific pathology, and one muscle biopsy with neurogenic changes (eTables S6 and S9). C5b‐9 deposition on capillaries and sarcolemma was noted in 58.8% and 47.2% of all ASS cases, respectively. Sarcoplasmic MxA expression was observed in three ASS patients (1.4%, 1 Jo‐1, 1 OJ, and 1 PL‐7‐positive patients) [21]; all of them were HLA‐DR‐negative. C5b‐9 and MxA expressions were not distinctively different among the ASS subtypes (eTable S7).

### 3.6 HLA‐DR expression was more common in IBM with patchy/diffuse and scattered patterns

HLA‐DR expression was much more common in IBM than in ASS (98.0% vs. 60.4%, *p* < 0.0001), with prominent patchy/diffuse (64.4%) followed by scattered (29.7%) patterns. Except for IBM, HLA‐DR expression was distinctively more common in ASS than in the other entities (14.9% DM, 6.4% IMNM, 0.7% P‐MM, *p* < 0.0001). Perifascicular HLA‐DR expression (pattern 3 + 4 + 5) is observed in 11.2% of DM (eTable S10); although the case number were limited, perifascicular HLA‐DR was common in anti‐SAE (2/5, 40.0%) and seronegative DM (33)(2/6, 33.3%). Notably, diffuse HLA‐DR expression was observed in one P‐MM (FSHD) patient; the muscle biopsy showed fibrofatty infiltration, macrophages infiltration, focal increased ALP activity in connective tissue, and HLA‐ABC and sarcolemmal MAC expression (eFigure S3).

### 3.7 Perifascicular HLA‐DR expression was highly specific for anti‐Jo‐1 ASS

Exclusion of entities potentially misdiagnosed as ASS by appropriate criteria (e.g., sarcoplasmic MxA expression for DM and clinicopathological criteria for IBM) help increase diagnostic performance of HLA‐DR to 95.4% specificity, 61.2% sensitivity, 85.9% PPV and 84.2% NPV. Perifascicular HLA‐DR expression (pattern 3 + 4 + 5) indicated a diagnosis of anti‐Jo‐1 ASS over the other entities with 94.9% specificity, 64.1% sensitivity, 61.2% PPV and 95.5% NPV (Table 2).

**TABLE 2:** Sensitivity, specificity, positive‐ and negative predictive value of HLA‐DR expression in ASS.

	ASS (*n* = 212)	Non‐ASS^a^ (*n* = 742)	Sensitivity	Specificity	PPV	NPV
HLA‐DR expression	128 (60.4)	148 (19.9)	60.4%	80.1%	46.4%	87.6%
Considering ASS versus other entities excluding MxA‐positive muscle biopsies and muscle biopsies from patient clinico‐pathologically compatible with IBM.						

	ASS^b^ (*n* = 209)	Non‐ASS^c^ (*n* = 453)	Sensitivity	Specificity	PPV	NPV
HLA‐DR expression	128 (61.2)	21 (4.6)	61.2%	95.4%	85.9%	84.2%
HLA‐DR 3 + 4 + 5	93 (44.5)	6 (1.3)	44.5%	98.7%	93.9%	79.4%
Considering anti‐Jo‐1 ASS versus other entities excluding MxA‐positive muscle biopsies and muscle biopsies from patient clinico‐pathologically compatible with IBM.						

	Jo‐1 ASS (*n* = 64)^d^	Non‐Jo‐1^c^ ^,^ ^e^ (*n* = 513)	Sensitivity	Specificity	PPV	NPV
HLA‐DR 3 + 4 + 5	41 (64.1)	26 (5.1)	64.1%	94.9%	61.2%	95.5%

*Note*: Pattern 1 and 1+ HLA‐DR expression show non‐specific localization. We did not include pattern 2 in “perifascicular pattern” because small number HLA‐DR‐positive myofibers were present in perifascicular areas; we suspected that such findings could be randomly present and non‐specific.

Abbreviations: ASS, antisynthetase syndrome; anti‐ARS, anti‐RNA synthetase; DM, dermatomyositis; HLA, human leukocyte antigen; IBM, inclusion body myositis; IMNM, immune‐mediated necrotizing myopathy; MxA, Myxovirus resistance protein A; NOS, not otherwise specify; NPV, negative predictive value; P‐MM, possible myositis mimics; PPV, positive predictive value.

^a^ Non‐ASS include DM = 188, IMNM = 313, IBM = 101, P‐MM = 140.

^b^ Exclude 3 MxA‐positive ASS: anti‐Jo‐1 = 1; anti‐OJ =1; anti‐PL‐7 = 1.

^c^ Exclude DM = 188 and IBM = 101.

^d^ Exclude 1 MxA‐positive anti‐Jo‐1 ASS.

^e^ Exclude 2 MxA‐positive ASS: anti‐OJ =1, anti‐PL‐7 = 1, exclude anti‐ARS_NOS.

### 3.8 IFN‐γ inducible genes were the most significantly upregulated IFN genes in ASS and IBM

The most significantly upregulated IFN genes in anti‐Jo‐1, anti‐OJ ASS, and IBM were the type II IFN (IFN‐ γ)‐inducible genes (e.g., *PSMB8* and *B2M*). Type I IFN (IFN1)‐inducible genes (e.g., *ISG15*, *IFI6, OAS* gene families) were highly upregulated in anti‐TIF1‐γ, Mi‐2, MDA5, and NXP‐2 DM. Anti‐HMGCR and anti‐SRP IMNM showed lower expression of these genes. Although the class II major histocompatibility complex transactivator (*CIITA*) was not present among the 10 most significantly upregulated IFN genes, its expression level in ASS and IBM showed a higher degree of significance than that of the other entities (eFigure S4, Table 3).

**TABLE 3:** Expression levels of the 10 most significantly expressed genes of the IFN‐signaling pathway and *CIITA* in different subtypes of inflammatory myopathy.

Jo‐1				OJ				IBM				HMGCR				SRP		
Gene	L2FC	Padj		Gene	L2FC	Padj		Gene	L2FC	Padj		Gene	L2FC	Padj		Gene	L2FC	Padj
*PSMB8*	3.75	2.62 E‐51		*PSMB8*	3.22	1.89 E‐38		*PSMB8*	3.44	4.79 E‐36		*IFI30*	3.89	7.25 E‐16		*IFI30*	3.83	1.59 E‐15
*B2M*	2.76	1.04 E‐35		*IFI30*	5.45	1.32 E‐35		*B2M*	2.56	6.97 E‐26		*FCGR1A*	4.65	3.36 E‐11		*TRIM14*	2.59	8.26 E‐12
*IRF9*	2.68	2.04 E‐29		*B2M*	2.47	1.73 E‐29		*HLA‐A*	2.63	2.16 E‐22		*NCAM1*	3.48	2.63 E‐10		*PSMB8*	1.76	2.38 E‐10
*IRF1*	3.51	7.17 E‐28		*TRIM14*	3.71	3.04 E‐27		*GBP6*	7.49	7.03 E‐22		*IRF5*	2.28	4.57 E‐10		*IRF9*	1.64	3.56 E‐10
*HLA‐A*	2.61	2.39 E‐26		*ICAM1*	3.24	2.14 E‐25		*HLA‐F*	2.88	4.48 E‐21		*TRIM14*	2.36	7.70 E‐10		*IRF5*	2.22	1.20 E‐09
*ICAM1*	3.35	2.94 E‐26		*FCGR1A*	6.54	5.99 E‐25		*HLA‐B*	3.11	9.45 E‐21		*OAS1*	2.49	1.37 E‐09		*OAS1*	2.47	1.74 E‐09
*GBP1*	4.19	5.63 E‐26		*IFI6*	3.89	1.89 E‐21		*HLA‐E*	1.84	5.18 E‐19		*IRF8*	2.79	4.69 E‐09		*IRF7*	1.96	3.22 E‐09
*HLA‐F*	2.92	9.82 E‐26		*SAMHD1*	2.06	5.92 E‐21		*HLA‐DRA*	3.68	1.55 E‐18		*TRIM38*	1.61	5.68 E‐09		*IRF8*	2.79	4.67 E‐09
*IFI30*	4.67	1.08 E‐25		*HLA‐A*	2.25	1.75 E‐20		*HLA‐DPA1*	3.38	1.64 E‐18		*OASL*	2.98	6.43 E‐09		*OAS2*	2.28	1.11 E‐08
*HLA‐B*	2.87	4.27 E‐21		*VCAM1*	3.56	2.00 E‐20		*HLA‐DPB1*	3.57	2.40 E‐18		*HLA‐DPA1*	2.19	1.46 E‐08		*VCAM1*	2.42	1.91 E‐08
*CIITA*	2.43	6.53 E‐13		*CIITA*	1.80	5.71 E‐08		*CIITA*	2.58	2.10 E‐12		*CIITA*	1.29	7.40 E‐04		*CIITA*	1.04	6.75 E‐03

TIF1‐γ				Mi‐2				MDA5				NXP‐2						
Gene	L2FC	padj		Gene	L2FC	padj		Gene	L2FC	padj		Gene	L2FC	padj				
*ISG15*	8.20	9.96 E‐63		*IFI6*	6.48	9.84 E‐54		*ISG15*	7.68	5.38 E‐66		*ISG15*	8.04	8.69 E‐93				
*IFI6*	7.13	2.93 E‐61		*OAS1*	5.51	4.26 E‐48		*IFI6*	6.70	8.76 E‐65		*IFI6*	6.74	4.24 E‐84				
*IRF9*	3.95	6.87 E‐57		*IFITM1*	3.68	2.38 E‐46		*IRF9*	3.85	8.76 E‐65		*IFIT3*	7.04	7.98 E‐81				
*IFIT3*	7.15	3.16 E‐54		*ISG15*	6.81	2.47 E‐46		*IFIT3*	6.85	1.28 E‐59		*OAS1*	5.90	1.20 E‐79				
*OAS1*	5.97	2.83 E‐53		*MX2*	4.60	1.08 E‐44		*OAS1*	5.55	5.88 E‐55		*IFITM1*	4.00	1.81 E‐79				
*RSAD2*	5.93	1.45 E‐48		*OAS3*	4.89	2.36 E‐43		*RSAD2*	5.62	5.70 E‐52		*IRF9*	3.65	6.74 E‐75				
*IFITM1*	3.78	3.35 E‐46		*IFI27*	4.27	2.08 E‐42		*IFIT5*	3.42	4.61 E‐50		*OAS3*	5.15	2.05 E‐69				
*OAS3*	5.20	4.08 E‐46		*OAS2*	4.91	1.10 E‐40		*OASL*	6.57	9.90 E‐50		*RSAD2*	5.67	3.57 E‐68				
*IFIT5*	3.55	3.55 E‐45		*IFI30*	5.93	2.77 E‐39		*OAS3*	4.85	5.79 E‐48		*IFIT5*	3.52	6.05 E‐68				
*PSMB8*	3.71	7.04 E‐45		*IFITM3*	3.31	4.15 E‐39		*IFITM1*	3.51	1.65 E‐47		*PSMB8*	3.69	8.89 E‐68				
*CIITA*	0.96	1.72 E‐02		*CIITA*	1.07	2.93 E‐03		*CIITA*	0.35	0.44		*CIITA*	1.02	7.67 E‐04				

Abbreviations: L2FC, log2 fold‐change; padj, adjusted *p* value.

## 4 DISCUSSION

This is a comprehensive myopathology study to describe features of different ASS subtypes. Because the commercially available antibody detection kit and ELISA likely fail to detect anti‐OJ‐antibodies due to their structural complexity, we used RNA immunoprecipitation for all anti‐OJ detection [15, 16, 20, 35]. Compared to the other ASS subtypes, the clinical and myopathological features of anti‐OJ ASS suggest more severe muscle involvement: higher CK level, shorter disease duration before biopsy, higher muscle fiber domain, and inflammatory domain scores. Although these results could be partly affected by antibody test bias in seven anti‐OJ cases, the results conform with the bias‐free original cohort [19, 20, 21] (eTables S11 and S12). Whether the more severe muscle involvement in anti‐OJ ASS affects overall disease prognosis compared to the other ASS subtype needs long term study preferably among the patients identified by immunoprecipitation or comparable method [36]. The lower CK levels and inflammatory domain score in anti‐PL‐12 ASS suggests milder muscle disease involvement [9]. Except for a lower number of patients with fever in anti‐Jo‐1 ASS, there was no difference in other extramuscular symptoms among the ASS subtypes.

Anti‐Jo‐1 ASS has been categorized as an immune myopathy with perimysial pathology (IMPP) showing a combination of PFN and perimysial pathology (i.e., perimysial connective tissue fragmentation and increased perimysial ALP activity) [13]. Nevertheless, these features are shared by other ASS subtypes [19] and anti‐Mi‐2 DM [11, 14]. The fact that PFN is more common in anti‐PL‐7 ASS could be explained by their myopathology severity which ranks between the higher end of the spectrum (represented by anti‐OJ and anti‐EJ ASS) and the lower end of the spectrum (represented by anti‐PL‐12 and anti‐Jo‐1 ASS). Notably, PFN is not present in our anti‐PL‐12 ASS. In our study, the most common myopathology pattern in ASS is necrotizing myopathy without PFN. Perimysial connective tissue fragmentation is not distinctively different among ASS subtypes. The more common perimysial ALP expression in anti‐OJ and anti‐EJ ASS is likely associated with the high perimysial CD68‐positive cell infiltration scores (eTable S5); a subset of tumor necrosis factor‐α and interleukin‐1β‐secreting CD68‐positive cells could induce tissue‐nonspecific ALPs expression [37, 38]. Reduction of CFR in all ASS subtypes, except for anti‐PL‐7, could be the result of C5b‐9 deposition capillary damage [29]. Interestingly, while the number of anti‐PL‐7 ASS with capillary C5b‐9 deposition in our study is not different from the other antibody subtypes, their CFR is not significantly decreased. These results suggest possible unidentified factor(s) causing different degree of capillary damage and tissue hypoxia among ASS subtypes. The higher number of PFA in anti‐EJ and anti‐OJ ASS could be attributed to their more profound muscle pathology. The underlying mechanism for tissue injury is possibly different considering the higher inflammatory domain score in anti‐OJ ASS. Whether the underlying pathomechanism(s) of ASS features are different from those of the features shared with other autoimmune myositis, especially DM, remains to be elucidated.

Major histocompatibility complex class I (MHC) and II are essential antigen presenting molecules in CD8 + T and CD4 + Tcell‐adaptive immune response. Human leukocyte antigens (HLA) are MHC molecules that each of them expressed from three highly pleomorphic gene regions (MHC class I: HLA‐A, HLA‐B, HLA‐C, and MHC class II: HLA‐DR, HLA‐DP, HLA‐DQ) [39, 40]. The proteins can be detected by immunohistochemistry for HLA‐ABC (MHC class I) and HLA‐DR (MHC class II); these proteins do not express on normal myofiber [17]. The master regulator of MHC class I and II expression are MHC class I transactivator (CITA/NLC5) and CIITA, respectively. While CITA/NLC5 can be induced by both type of IFN, CIITA is mainly induced by type 2 IFN [39, 40]. Unlike HLA‐DR, HLA‐ABC is more commonly expressed in various muscle diseases [17, 18].

Smaller studies previously addressed the pattern of the HLA‐ABC, HLA‐DR, and C5b‐9 expression in ASS [10, 29]. We observe HLA‐ABC expression in 92.5% of ASS. Perifascicular HLA‐ABC enhancement is more common in anti‐Jo‐1 ASS but at a lower percentage than the previous study (26.2% vs.79%) [29]. In a French ASS study which 88% (44/50) of the cases were anti‐Jo‐1 ASS, HLA‐DR expression was reported in 82.8% of the ASS cases and all of them show perifascicular expression pattern [10]. In a recent study involving 10 muscle biopsies from anti‐Jo‐1, 6 from anti‐PL‐7, and 8 from anti‐PL‐12 ASS patients, the authors demonstrated similar pathological features among these ASS subtypes including PFN, MHC class I upregulation and prominent perifascicular MHC class II expression; only one PL‐12 ASS had negative MHC class II expression [41]. In line with the pathological features, the proteomic profiling of muscle associated with these three antibodies show no significant difference [ 41]. Unlike these cohorts, our study shows a smaller proportion of overall and perifascicular HLA‐DR expression. After exclusion of DM and IBM, the major entities that can disguise clinically and pathologically as ASS, and MxA‐positive ASS which may contain concurrent unidentified DMSA, the specificity of HLA‐DR expression for ASS increase to 95.4%. In this setting, presence of perifascicular HLA‐DR expression (pattern3 + 4 + 5) is highly specific (94.9%) to anti‐Jo‐1 ASS. Presence of IFN‐γ inducible genes as the most significantly upregulated IFN genes in ASS and IBM suggests IFN‐γ signaling pathway activation on CIITA which induce myofiber HLA‐DR expression in these diseases. Co‐expression of CIITA and HLA‐DR on perifascicular fibers also reflects activation of the IFN‐γ signaling pathway (eFigure S5) [40]. Among major subtypes of autoimmune myositis, IFN‐γ‐related genes are reported to be upregulated in IBM and ASS [42]. Although IFN‐γ can be produced by various types of inflammatory cells, presence of HLA‐DR expression in 10 biopsies with normal/nonspecific muscle pathology raising the possibility of other regulated mechanisms yet to be identified in ASS [10, 40]. TRI, the IFN1‐induced structure, are less commonly present in ASS than DM [43]. The finding is well‐correlated with a lower expression level of IFN1‐related genes in ASS [42] and the presence of scattered faint MxA‐positive capillaries in a subset of our ASS patients (60.9%, 98/161 unpublished data) but rare sarcoplasmic expression. Both sarcolemma and capillary C5b‐9 depositions are present in ASS and DM but strong capillary C5b‐9 depositions similar to those appear in DM are uncommon in ASS [11].

Considering clinical combination of muscle weakness accompanying with skin lesions and/or other extramuscular symptoms, the major differential diagnoses for ASS should include DM, IMNM and overlap myositis (OM, overlap syndrome) [44]. While anti‐Mi‐2 DM share perifasciular and perimysial pathology with ASS [11], DM associated with other DMSA subtypes show distinct pathological features: anti‐TIF1‐γ DM with vacuolated/punched out fiber; anti‐MDA5 DM with near normal or less muscle pathology and inflammatory features and anti‐NXP‐2 DM with microinfarction [43]. Anti‐SAE DM and seronegative DM tend to be associated with HLA‐DR expression [43]. In the absence of serological information, presence of sarcoplasmic MxA expression support the diagnosis of DM over the other entities [21, 45]. Extramuscular involvement is more common in anti‐SRP than anti‐HMGCR IMNM [44, 46]. Without serology test or immunohistochemical study, muscle biopsy in IMNM could be indistinguishable from ASS. In our study, presence of HLA‐DR expression favors the diagnosis of ASS over IMNM. Coexistence of myositis and another autoimmune disorder including but may not limited to systemic sclerosis, systemic lupus erythematosus, and rheumatoid arthritis defines OM. OM may or may not associated with autoantibodies including anti‐U1RNP, anti‐Ku, anti‐PM‐Scl, anti‐RuvBL, anti‐RuvBL2, anti‐Ro/SS‐A, and anti‐La/SS‐B. The clinical history and pathology findings in OM are heterogenous. Pathological findings can range from perimysial fibrosis with endothelial lesion and perifascicular ischemia to dermatomyositis‐like features and intense fibrosis and necrosis [44, 47, 48, 49]. HLA‐DR expression is reported in some OM [50]; perifascicular pattern has not yet identified.

Due to the retrospective nature of this study, the clinical information is limited and does not represent the prevalence/prognosis of muscular and extramuscular involvement throughout the disease course. All patients in this study underwent muscle biopsy for diagnostic purposes. Thus, the clinical information may also be biased omitting ASS patients without obvious muscle symptoms. All 15 HLA‐ABC negative and one HLA‐ABC negative but HLA‐DR positive ASS patients presented with muscle weakness. Considering patchy muscle involvement in ASS, these results could be false negative due to non‐representative biopsy sampling and/or tissue artifacts. We cannot prove whether the three ASS patients with MxA‐positive myofibers had concurrent unknown DMSA or had a higher IFN1 level than the other cases. Moreover, we cannot comment on a single case of anti‐KS ASS; anti‐Ha and anti‐Zo ASS is not present in our study. P‐MM pathology have been reported in FSHD, dysferlinopathy, sarcoglycanopathy, laminopathy, ANO5 myopathy, and FKRP myopathy [22]. In this study, entities associated with P‐MM pathology were evaluated for HLA‐DR expression regardless of their pathological patterns. In FSHD patient, disrepression DUX4 and DUX4 signaling associated with inflammatory response are likely associated with P‐MM pathology [51, 52]. However, the precise mechanism for HLA‐DR expression and its role in P‐MM pathology need further study; the process may differ among these P‐MM associated entities.

In appropriate clinicopathological context, absence of MxA but HLA‐DR expression can help differentiate ASS from the other major subtypes of autoimmune myositis and P‐MM even when serological information is limited. In addition, pathological features can help distinguish anti‐OJ from the other ASS subtypes when the anti‐ARS antibody assay result is incomplete or subjected to false negative. Further correlation of serum IFN‐γ levels and myofiber HLA‐DR expression and the potential benefits of IFN‐γ pathway inhibition in ASS remain to be determined.

## AUTHOR CONTRIBUTIONS

**JantimaTanboon:** Study concept and design; acquisition of data; analysis orinterpretation of data; drafting/revision of the manuscript for content, including medical writing for content. **Michio Inoue:** Acquisition of data; drafting/revision of the manuscript for content, including medical writing for content. **Shinya Hirakawa:** Drafting/revision of the manuscript for content, including medical writing for content. **Hisateru Tachimori:** Drafting/revision of the manuscript for content, including medical writing for content. **Shinichiro Hayashi:** Drafting/revision of the manuscript for content, including medical writing for content. **Satoru Noguchi:** drafting/revision of the manuscript for content, including medical writing for content. **Naoko Okiyama:** Acquisition of data; drafting/revision of the manuscript for content, including medical writing for content. **Manabu Fujimoto:** Acquisition of data; drafting/revision of the manuscript for content, including medical writing for content. **Shigeaki Suzuki:** Acquisition of data; drafting/revision of the manuscript for content, including medical writing for content. **Ichizo Nishino:** Study concept anddesign; acquisition of data; analysis or interpretation of data; drafting/revision of the manuscript for content, including medical writing for content.

## FUNDING INFORMATION

This study was supported by an Intramural Research Grant (2–5) for Neurological and Psychiatric Disorders of the NCNP and partly supported by AMED under the Grant number JP21ek0109490h0002.

## CONFLICT OF INTEREST STATEMENT

The authors report no conflict of interests.

## Supporting information


**Data S1:** Supporting Information.

## Data Availability

Anonymized data not published in this article will be made available upon request by any qualified investigator.
