# Supplementary material for: Muscle pathology of antisynthetase syndrome according to antibody subtypes
Source: Brain Pathol. 2023 Mar 7;33(4):e13155. doi: 10.1111/bpa.13155 (PMC10307527; doi:10.1111/bpa.13155)
Supplement: Supplementary file 1 — Data S1: Supporting Information. [file BPA-33-e13155-s001.pdf]

Supplementary eTable 1

Immunohistochemical stains used in this study

Immunohistochemical stain	Abbreviation used	Clone	Company
HLA-ABC	HLA-ABC	W6/32	Thermo Fisher Scientific
HLA-DR	HLA-DR	B308	Affinity Bioreagents
Membrane attack complex	C5b-9	aE11	Dako
Myxovirus resistance protein A	MxA	Mx 1/2/3	Santa Cruz Biotechnology
Neonatal myosin heavy chain	MHCn	WB-MHCn	Leica
Utrophin	Utrophin	DRP3/20C5	Novocastra
CD3	CD3	Polyclonal	Abcam
CD8	CD8	DK25	Dako
CD20	CD20	L26	eBioscience
CD68	CD68	KP1	Dako
p62	p62	SQSTM1	Santa Cruz Biotechnology
Dysferlin	Dysferlin	HamI/7B6	Leica
α–sarcoglycan	α–sarcoglycan	AdI/20A6	Leica
β–sarcoglycan	β–sarcoglycan	β–sarc/5B1	Leica
γ–sarcoglycan	γ–sarcoglycan	35DAG/21B5	Leica
Class II major histocompatibility complex transactivator	CIITA	7-1H	Santa Cruz Biotechnology

Abbreviations: HLA, human leukocyte antigen; CD, cluster of differentiation

Supplementary eTable 2

Pathology domains and scoring/evaluation criteria used for muscle biopsy assessment

Pathology domain	Scoring/evaluation criteria
**Muscle fiber domain**<br>Necrotic fiber	Maximum score = 8<br>0 = absence, 1 = isolated/sporadic 1–3 per 20x, 2 = scattered ≥4 or more in adjacent fibers, or clustered, or >3 per 20x
Regenerating fiber	0 = <6 per 20x, 1 = ≥6 per 20x
Atrophic fibers away from perifascicular area	0 = absence, 1 = isolated/sporadic 1–3 per 20x, 2 = scattered ≥ 4 or more in adjacent fibers, or clustered, or >3 per 20x
Perifascicular atrophy	0 = absence, 1 = affecting 1-2 fascicle(s), 2 = affecting ≥ 2 fascicles
Fiber with internalized nuclei >3%	0 = ≤3%, 1 = >3%
**Inflammatory domain**<br>Endomysial and perimysial CD3+ cell infiltration<br>Endomysial and perimysial CD20+ cell infiltration<br>Endomysial and perimysial CD68+ cell infiltration	Maximum score = 13<br>0 = none or <4 cells in 20x field, 1 = 4–20 cells in a 20x field or a cluster of ≥10 cells, 2 = ≥2 clusters in whole biopsy and/or diffusely infiltrating i.e. >20 cells in a 20x field (separate scores for endomysial and perimysial infiltration)
Perivascular inflammatory cell infiltration	0 = absence, 1 = presence
**Vascular domain**<br>Capillary:fiber ratio	Utrophin-positive capillaries per the number of muscle fibers in 10 randomly photographed areas at 20x or at least 5 areas at 20x in the case of small biopsy
**Connective tissue domain**<br>Perimysial connective tissue fragmentation	N = absence, Y = presence
Increased alkaline phosphatase activity in perimysium	N = absence, Y = presence
Endomysial fibrosis	N = absence, Y = presence

Abbreviations: N = no; Y= yes

Supplementary eTable 3

Pathology domains in antisynthetase syndrome

	Anti-ARS antibody							
	**All ARS** (n = 212)	**Jo-1** (n = 65)	**OJ** (n = 20)	**PL-7** (n = 20)	**PL-12** (n = 11)	**EJ** (n = 10)	**KS** (n = 1)	**ARS_NOS** (n = 85)
**Muscle fiber domain**								
Score	3.0±1.8	2.5±1.4*	4.6±2.0*	3.1±2.1	2.5±1.9	4.7±2.1*	4.0	2.8±1.7
**Inflammatory domain**								
Score	4.9±3.0[a]	4.9±3.2[b]	6.8±3.2*	4.2±1.9	2.6±1.5[c,]*	4.5±3.0	2.0	5.0±3.0[b]
**Vascular domain**								
Capillary: myofiber ratio	0.8±0.4[d]	0.9±0.5[c]	0.8±0.2[b]	0.9±0.5[b]	0.8±0.3	0.6±0.3*	0.8	0.8±0.4[e]
**Connective tissue domain**								
PM-Fr	109 (51.9)[c]	29 (44.6)	14 (70.0)	11 (55.0)	5 (45.5)	7 (60.0)	0	44 (53.0)[c]
PM-ALP	102 (48.1)	22 (33.8)*	14 (70.0)*	10 (50.0)	3 (27.3)	9 (90.0)*	1	43 (50.6)
Endomysial fibrosis	34 (16.2)[c]	5 (7.7)*	4 (20.0)	2 (10.0)	3 (27.3)	6 (60.0)*	0	14 (16.9)[c]

Continuous data is shown as mean and ± standard deviation while categorial data is reported as number and (percentage).
Abbreviations: ARS, anti-tRNA synthetase; NOS, not otherwise specified; PM-Fr, perimysial fragmentation; PM-ALP, increased perimysial alkaline phosphatase activity.
[a]Four cases were excluded from the analysis due to artifacts
[b]One case was excluded from the analysis due to artifacts
[c]Two cases were excluded from the analysis due to artifacts
[d]Seven cases were excluded from the analysis due to artifacts
eThree cases were excluded from the analysis due to artifacts
*p < 0.05 compared to the other antibody subtypes

Supplementary eTable 4

Muscle fiber domain scores in antisynthetase syndrome

	Anti-ARS antibody							
	**All ARS** (n = 212)	**Jo-1** (n = 65)	**OJ** (n = 20)	**PL-7** (n =20)	**PL-12** (n = 11)	**EJ** (n = 10)	**KS** (n = 1)	**ARS_NOS** (n = 85)
**Muscle fiber domain**								
Total Score	3.0±1.8	2.5±1.4*	4.6±2.0*	3.1±2.1	2.5±1.9	4.7±2.1*	4.0	2.8±1.7
Necrotic fiber score	1.1±0.8	0.9±0.7*	1.7±0.6*	1.3±0.6	0.7±0.8	1.3±0.8	2.0	2.5±1.4
Regenerating fiber score	0.6±0.5	0.6±0.5	0.9±0.4*	0.6±0.5	0.4±0.5	0.8±0.4	1.0	0.6±0.5
Atrophic fiber score	0.7±0.8	0.6±0.7*	1.0±0.9	0.7±0.9	0.8±0.9	1.2±0.8	0	0.7±0.8
PFA score	0.2±0.6	0.1±0.4*	0.7±0.9*	0.3±0.7	0.3±0.6	0.8±0.8	1.0	0.2±0.5
Fiber with internalized nuclei score	0.3±0.5	0.3±0.5	0.3±0.5	0.2±0.4	0.3±0.5	0.6±0.5	0	0.2±0.4
Fiber with internalized nuclei >3%	60 (28.3)	22 (33.8)	6 (30.0)	4 (21.1)	3 (27.3)	6 (54.5)	0	19 (22.4)

Continuous data is shown as mean and ± standard deviation while categorial data is reported as number and (percentage).
Abbreviations: ARS, anti-tRNA synthetase; NOS, not otherwise specified; PFA, perifascicular atrophy
*p < 0.05 compared to the other antibody subtypes

Supplementary eTable 5

Inflammatory domain in antisynthetase syndrome

	Anti-ARS antibody							
	**All ARS** (n = 212)	**Jo-1** (n = 65)	**OJ** (n = 20)	**PL-7** (n = 20)	**PL-12** (n = 11)	**EJ** (n = 10)	**KS** (n = 1)	**ARS_NOS** (n = 85)
**Inflammatory domain**								
Total Score	4.9±3.0[a]	4.9±3.2[b]	6.8±3.2*	4.2±1.9	2.6±1.5[c,*]	4.5±3.0	2.0	5.0±3.0[b]
Endomysial CD3 infiltration	0.7±0.8[a]	0.8±0.8[b]	1.1±0.9	0.8±0.5	0.1±0.3[c,*]	0.7±0.8	0	0.7±0.7[b]
Perimysial CD3 infiltration	0.3±0.6[a]	0.3±0.7[b]	0.6±0.7	0*	0[c,*]	0.2±0.4	0	0.3±0.6[b]
Endomysial CD20 infiltration	0.4±0.7[a]	0.5±0.7[b]	0.7±0.7	0.4±0.6	0.1±0.3[c,*]	0.2±0.4	0	0.4±0.7[b]
Perimysial CD20 infiltration	0.2±0.5[a]	0.2±0.5[b]	0.3±0.6	0.2±0.4	0[c,*]	0.1±0.3	0	0.3±0.6[b]
Endomysial CD68 infiltration	1.6±0.5[a]	1.6±0.6[b]	1.9±0.4*	1.7±0.5	1.3±0.7[c]	1.7±0.7	0	1.6±0.5[b]
Perimysial CD68 infiltration	1.1±0.8[a]	1.0±0.8[b]	1.6±0.7*	0.8±0.8	0.8±0.7[c]	1.2±0.9	0	1.3±0.7[b]
Perivascular inflammatory cell infiltration score	0.4±0.5	0.4±0.5	0.8±0.4*	0.4±0.5	0.2±0.4	0.4±0.5	0	0.4±0.5
Perivascular inflammatory cell infiltration	87 (41.0)	26 (40.0)	15 (75.0)*	7 (35.0)	2 (18.2)	4 (40.0)	0	33 (38.8)

Categorial data is reported as number and (percentage).
Abbreviations: ARS, anti-tRNA synthetase; NOS not otherwise specified; CD, cluster of differentiation
[a]Four cases were excluded from the analysis due to artifacts
[b]One case was excluded from the analysis due to artifacts
[c]Two cases were excluded from the analysis due to artifacts
*p < 0.05 compared to the other antibody subtypes

Supplementary eTable 6

Myopathology patterns and histological features of interest in antisynthetase syndrome

	Anti-ARS antibody							
	**All ARS** (n = 212)	**Jo-1** (n = 65)	**OJ** (n = 20)	**PL-7** (n = 20)	**PL-12** (n = 11)	**EJ** (n = 10)	**KS** (n = 1)	**ARS_NOS** (n = 85)
**Myopathology patterns**[a]								
Normal/non-specific	37 (17.6)	13 (20.0)	0*	2 (10.0)	3 (27.3)	2 (20.0)	0	17 (20.5)
Necrotizing myopathy without PFN	97 (46.2)	25 (38.5)	10 (50.0)	7 (35.0)	6 (54.5)	5 (50.0)	0	44 (53.0)
Necrotizing myopathy with PFN	59 (28.1)	19 (29.2)	9 (45.0)	11 (55.0)*	0*	3 (30.0)	1 (100.0)	16 (19.3)
Others	17 (8.1)	8 (12.3)[b,c]	1 (5.0)[d]	0	2 (18.2)[e]	0	0	6 (7.2)[f]
**Histological features of interest**								
PFN	60 (28.3)	20 (30.8)	9 (45.0)	11 (55.0)*	0*	3 (30.0)	1 (100.0)	16 (18.8)
PFA	36 (17.0)	6 (9.2)*	8 (40.0)*	3 (15.0)	2 (18.2)	6 (60.0)*	1 (100.0)	10 (11.8)
PF-COX, decreased	9 (4.2)	2 (3.1)	2 (10.0)	1 (5.0)	0	1 (10.0)	0	3 (3.5)
Vasculitis[g]	36 (17.0)	8 (12.3)	9 (45.0)*	3 (15.0)	0	1(10.0)	0	15 (17.6)
CD8 infiltration in non-necrotic fiber	1 (0.5)	0	0	0	0	0	0	1 (1.2)
CD68/ACP infiltration in non-necrotic fiber	7 (3.3)	2 (3.1)	1 (5.0)	1 (5.3)	0	1 (9.1)	0	2 (2.4)
CD20 aggregation	10 (4.7)	4 (6.2)	1 (5.0)	1(5.3)	0	0	0	4 (4.7)

Categorial data is reported as number and (percentage). *$p < 0.05$ compared to the other antibody subtypes
Abbreviations: ARS, anti-tRNA synthetase; ACP, acid phosphatase; CD, cluster of differentiation; COX, cytochrome C oxidase; PF, perifascicular; PFA, perifascicular atrophy; PFN, perifascicular necrosis; NOS, not otherwise specified
[a] Two cases of ARS, nos were excluded from the analysis due to artifacts
[b]One case classified as granulomatous myositis showed concurrent PFN and granulomatous inflammation
[c]Type 2 fiber atrophy (1), Neurogenic change (4), rimmed vacuole (1), nemaline bodies (1)
[d]Type 2 fiber strophy (1)
[e]Type 2 fiber strophy (1), rimmed vacuole (1)
[f]Neurogenic change (2), rimmed vacuole (2), nemaline bodies (1), cytoplasmic body (1)
[g]Vasculitis: presence of inflammatory cells infiltratation in the vascular wall

Supplementary eTable 7

Immunohistochemical feature in antisynthetase syndrome

	Anti-ARS antibody							
	**All ARS** (n = 212)	**Jo-1** (n = 65)	**OJ** (n = 20)	**PL-7** (n = 20)	**PL-12** (n = 11)	**EJ** (n = 10)	**KS** (n = 1)	**ARS_NOS** (n = 85)
HLA-ABC expression	196 (92.5)	6 1(93.8)	20 (100.0)	20 (100.0)	9 (81.8)	10 (100.0)	1 (100.0)	75 (88.2)
HLA-ABC expression with PF enhancement	36 (17.0)	17 (26.2)*	1 (5.0)	0	0	1 (10.0)	0	17 (20.0)
HLA-DR expression	128 (60.4)	44 (67.7)	13 (65.0)	10 (50.0)	5 (45.5)	5 (50.0)	1 (100.0)	50 (58.8)
C5b-9 capillary deposition	124 (58.8)[a]	42 (64.6)	10 (50.0)	13 (65.0)	6 (54.5)	4 (40.0)	0	49 (58.3)[a]
C5b-9 capillary deposition in PF area	13 (6.2)[a]	6 (9.2)	1 (5.0)	0	2 (18.2)	0	0	4 (4.8)
C5b-9 sarcolemmal deposition	100 (47.2)	27 (41.5)	10 (50.0)	8 (40.0)	2 (18.2)	8 (80.0)*	0	45 (52.9)
C5b-9 sarcolemmal deposition in PF area	64 (30.2)	19 (29.2)	7 (35.0)	4 (20.0)	1 (9.1)	6 (60.0)	0	27 (31.8)
MxA expression	3 (1.4)	1 (1.5)	1 (5.0)	1 (5.0)	0	0	0	0

Categorial data is reported as number and (percentage).
Abbreviations: ARS, anti-tRNA synthetase; HLA, human leukocyte antigen; NOS, not otherwise specified; C5b-9, membrane attack complex; MxA, Myxovirus resistance protein A; PF, perifascicular
[a]One case was not included due to artifacts
*$p < 0.05$ compared to the other antibody subtypes

Supplementary eTable 8

HLA-DR expression patterns in ASS

	Anti-ARS antibody							
	**All ARS** (n = 212)	**Jo-1** (n = 65)	**OJ** (n = 20)	**PL-7** (n = 20)	**PL-12** (n = 11)	**EJ** (n = 10)	**KS** (n = 1)	**ARS_NOS** (n = 85)
HLA-DR expression	128 (60.4)	44 (67.7)	13 (65.0)	10 (50.0)	5 (45.5)	5 (50.0)	1 (100.0)	50 (58.8)
Pattern 1	19 (9.0)	1 (1.5)*	2 (10.0)	1 (5.0)	3 (27.3)*	2 (20.0)	0	10 (11.8)
Pattern 1+	3 (1.4)	1 (1.5)	1 (5.0)	0	0	0	0	1 (1.2)
Pattern 2	13 (6.1)	1 (1.5)*	3 (15.0)	2 (10.0)	0	0	0	7 (8.2)
Pattern 3	14 (6.6)	2 (3.1)	1 (5.0)	4 (20.0)*	0	0	1 (100.0)	6 (7.1)
Pattern 4	60 (28.3)	30 (46.2)*	4 (20.0)	3 (15.0)	2 (18.2)	2 (10.0)	0	19 (22.4)
Pattern 5	19 (9.0)	9 (13.8)	2 (10.0)	0	0	1 (10.0)	0	7 (8.2)
Perifascicular pattern (3+4+5)	93 (43.9)	41 (63.1)*	7 (35.0)	7 (35.0)	2 (18.2)	3 (30.0)	1 (100.0)	32 (37.6)

Categorial data is reported as number and (percentage).
Note: Pattern 1 and 1+ HLA-DR expression show non-specific localization. We did not include pattern 2 in "perifascicular pattern" because small number HLA-DR-positive myofibers were present in perifascicular areas; we suspected that such findings could be randomly present and non-specific.
Abbreviations: ARS, anti-tRNA synthetase; HLA, human leukocyte antigen; NOS, not otherwise specified
*$p < 0.05$ compared to the other antibody subtypes

Supplementary eTable 9

Myopathology patterns and HLA-DR expression pattern 3, 4, and 5

	Anti-ARS antibody							
	**All ARS** (n = 210)[a]	**Jo-1** (n = 65)	**OJ** (n = 20)	**PL-7** (n = 20)	**PL-12** (n = 11)	**EJ** (n = 10)	**KS** (n = 1)	**ARS_NOS** (n = 83)[a]
**HLA-DR expression**								
Normal/non-specific	10 (4.8)	4 (6.2)	0	0	0	1 (10.0)	0	5 (6.0)
Necrotizing myopathy without PFN	63 (30.0)	18 (27.7)	7 (35.0)	5 (25.0)	3 (27.3)	1 (10.0)	0	29 (34.9)
Necrotizing myopathy with PFN	49 (23.3)	19 (29.2)	6 (30.0)	5 (25.0)	0	3 (30.0)	1 (100)	15 (18.1)
Others	5 (2.4)	3 (4.6)	0	0	2 (18.2)	0	0	0
**Pattern 3**								
Normal/non-specific	3 (1.4)	1 (1.5)	0	0	0	0	0	1 (1.2)
Necrotizing myopathy without PFN	5 (2.4)	1 (1.5)	0	3 (15.0)	0	0	0	1 (1.2)
Necrotizing myopathy with PFN	6 (2.9)	0	1 (5.0)	1 (5.0)	0	0	1 (100)	3 (3.6)
Others	0	0	0	0	0	0	0	0
**Pattern 4**								
Normal/non-specific	6 (2.9)	3(4.6)	0	0	0	0	0	3 (3.6)
Necrotizing myopathy without PFN	29 (13.8)	14 (21.5)	2 (10.0)	1 (5.3)	2 (18.2)	0	0	10 (12.0)
Necrotizing myopathy with PFN	24 (11.4)	12 (18.5)	2 (10.0)	2 (10.5)	0	2 (18.2)	0	6 (7.2)
Others	1 (0.5)	1 (1.5)[b]	0	0	0	0	0	0
**Pattern 5**								
Normal/non-specific	1 (0.5)	0	0	0	0	1 (9.1)	0	0
Necrotizing myopathy without PFN	7 (3.3)	2 (3.1)	1 (5.0)	0	0	0	0	4 (4.8)
Necrotizing myopathy with PFN	11 (5.2)	7 (10.8)	1 (5.0)	0	0	0	0	3 (3.6)
Others	0	0	0	0	0	0	0	0

Categorial data is reported as number and (percentage). *$p < 0.05$ compared to the other antibody subtypes
Abbreviations: ARS, anti-tRNA synthetase; NOS, not otherwise specified; PFN, perifascicular necrosis
[a]Two cases of ARS_NOS were excluded from the analysis due to artifacts
[b]Neurogenic muscle biopsy

Supplementary eTable 10

HLA-DR expression patterns in non-ASS AIM and P-MM

	Non-ASS AIM (n = 602)			P-MM (n = 140)					
	**DM** (n = 188)	**IMNM** (n = 313)	**IBM** (n = 101)	**DYSF** (n = 50)	**SGP** (n = 15)	**LMNA** (n = 16)	**ANO5** (n = 3)	**FKRP** (n = 9)	**FSHD** (n = 47)
HLA-DR expression	28 (14.9)*	20 (6.4)*	99 (98.0)*	0	0	0	0	0	1 (2.1)*
Pattern 1	3 (1.6)	11 (3.5)	30 (29.7)	0	0	0	0	0	1 (2.1)
Pattern 1+	0	0	65 (64.4)	0	0	0	0	0	0
Pattern 2	4 (2.1)	3 (1.0)	1 (1.0)	0	0	0	0	0	0
Pattern 3	7 (3.7)	2 (0.6)	0	0	0	0	0	0	0
Pattern 4	10 (5.3)	4 (1.3)	0	0	0	0	0	0	0
Pattern 5	4 (2.1)	0	3 (3.0)	0	0	0	0	0	0
Perifascicular pattern (3+4+5)	21 (11.2)	6 (1.9)	3 (3.0)	0	0	0	0	0	0

Categorial data is reported as number and (percentage).
Note: Pattern 1 and 1+ HLA-DR expression show non-specific localization. We did not include pattern 2 in "perifascicular pattern" because small number HLA-DR-positive myofibers were present in perifascicular areas; we suspected that such findings could be randomly present and non-specific.
Abbreviations: AIM, autoimmune myositis; ASS, antisynthetase syndrome; P-MM, possible myositis mimics; DM, dermatomyositis; IMNM, immune-mediated necrotizing myopathy; IBM, inclusion body myositis; DYSF, dysferlinopathy; SGP, sarcoglycanopathy; LMNA, laminopathy; ANO5, anoctamin5 myopathy; FKRP, fukutin-related protein myopathy; FSHD, facioscapulohumeral muscular dystrophy
*$p < 0.05$ compared to ASS

Supplementary eTable 11

Pathology domains in antisynthetase syndrome of the original cohort

	Anti-ARS antibody						
	**All ARS** (n = 50)	**Jo-1** (n = 15)	**OJ** (n = 13)	**PL-7** (n = 12)	**PL-12** (n = 4)	**EJ** (n = 5)	**KS** (n = 1)
**Muscle fiber domain**							
score	3.3±2.2	2.2±1.3*	5.2±2.0*	3.3±2.5	1.3±0.5*	3.6±2.4	4.0
**Inflammatory domain**							
score	4.5±2.9	3.9±2.9	7.2±3.0*	3.9±1.8	2.8±1.0*	2.8±2.4	2.0
**Vascular domain**							
Capillary: muscle fiber ratio	0.8±0.3[a]	0.9±0.5	0.9±0.2[b]	0.8±0.4[b]	0.7 ±0.1	0.7 ±0.2	0.8
**Connective tissue domain**							
PM-Fr	27 (54.0)	6 (40)	9 (69.2)	7 (58.3)	3 (75.0)	2 (40.0)	0
PM-ALP	29 (58.0)	6 (40)	10 (76.9)	8 (66.7)	0*	4 (80.0)	1 (100.0)
Endomysial fibrosis	3 (6.0)	0	1 (7.7)	1 (8.3)	0	1 (20.0)	0

Continuous data is shown as mean and ± standard deviation while categorial data is reported as number and (percentage).
Abbreviations: ARS, anti-tRNA synthetase; PM-Fr, perimysial connective tissue fragmentation; PM-ALP, increased perimysial alkaline phosphatase activity
[a]Two cases were excluded from analysis due to artifacts
[b]One case was excluded from analysis due to artifacts

Supplementary eTable 12

Immunohistochemical feature in antisynthetase syndrome of the original cohort

	Anti-ARS antibody						
	**All ARS** (n = 50)	**Jo-1** (n = 15)	**OJ** (n = 13)	**PL-7** (n = 12)	**PL-12** (n = 4)	**EJ** (n = 5)	**KS** (n = 1)
HLA-ABC expression	49 (98.0)	14 (93.3)	13 (100.0)	12 (100.0)	4 (100.0)	5 (100.0)	1 (100.0)
HLA-ABC expression with PF enhancement	2 (4.0)	2 (13.3)	0	0	0	0	0
HLA-DR expression	27 (54.0)	9 (60.0)	8 (61.5)	4 (33.3)	2 (50.0)	3 (60.0)	1 (100.0)
HLA-DR expression with perifascicular pattern (3+4+5)	20 (40.0)	8 (53.3)	5 (38.5)	2 (16.7)	2 (50.0)	2 (40.0)	1 (100.0)
C5b-9 capillary deposition	28 (56.0)	12 (80.0)*	7 (53.8)	5 (41.7)	2 (50.0)	2 (40.0)	0
C5b-9 capillary deposition in PF area	2 (4.0)	1(6.7)*	1 (7.7)*	0*	0*	0*	0
C5b-9 sarcolemmal deposition	19 (38.0)	4 (26.7)	7 (53.8)	5 (41.7)	0	3 (60)	0
C5b-9 sarcolemmal deposition in PF area	14 (28.0)	2 (13.3)	6 (46.2)	4 (33.3)	0	2 (40.0)	0
MxA expression	1 (2.0)	0	0	1 (8.3)	0	0	0

Continuous data is shown as mean and ± standard deviation while categorial data is reported as number and (percentage).
Abbreviations: ARS, anti-tRNA synthetase; HLA, human leukocyte antigen; PF, perifascicular area; C5b-9, membrane attack complex; MxA, myxovirus resistant protein A
*$p < 0.05$ compared to the other antibody subtype

Supplementary eFigure 1

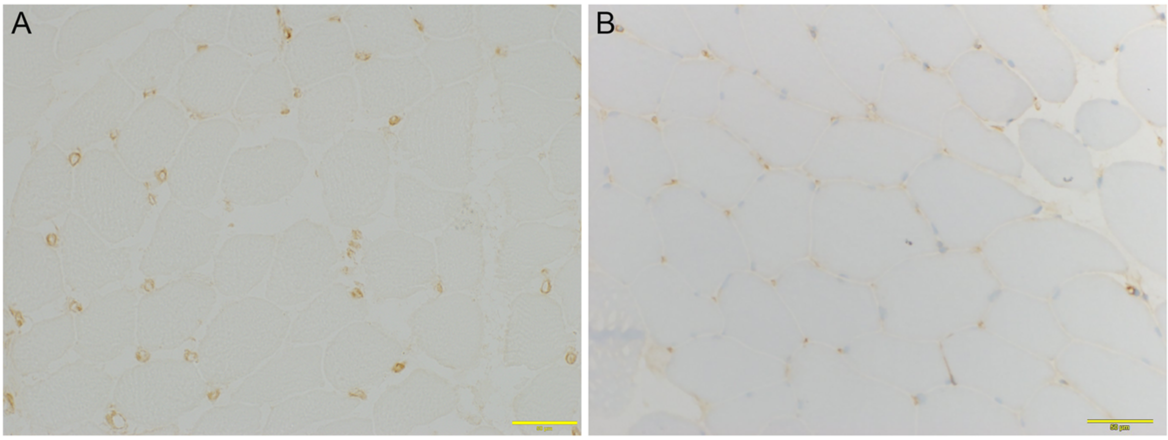

Representative figure showing decreased capillary: myofiber ratio: The number of utrophin-positive capillaries is focally decreased in this anti-OJ ASS (A) compared to the control (B).

Note: bar = 50 μm; Utrophin

Supplementary eFigure 2.

Representative figure showing HLA-ABC staining pattern

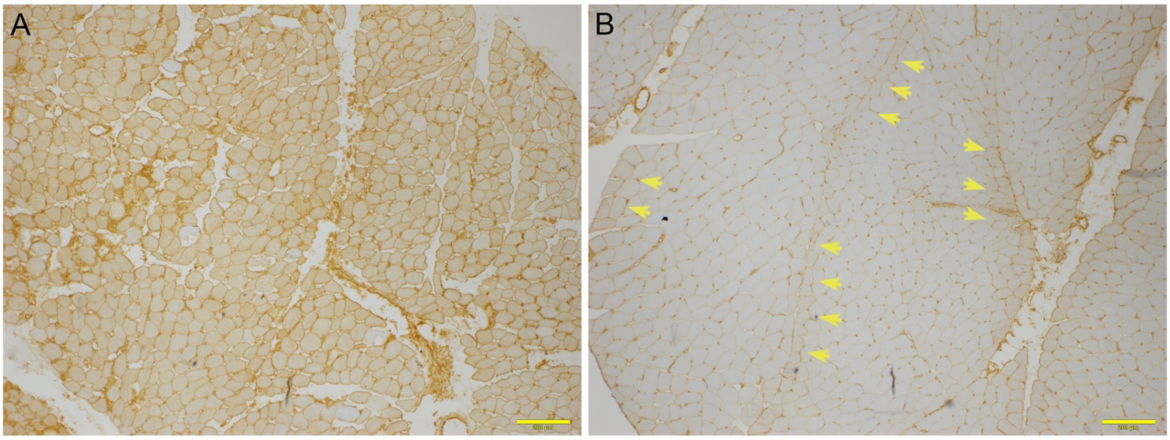

Representative figure showing HLA-ABC staining pattern: This anti-OJ ASS shows diffuse HLA-ABC expression pattern (A) while this anti-Jo-1 ASS shows HLA-ABC expression with perifascicular enhancement (B, arrows)

Note: bar = 200 μm; HLA-ABC, human leukocyte antigen-ABC, major histocompatibility complex class I.

Supplementary eFigure 3.

P-MM: FSHD with HLA-DR expression

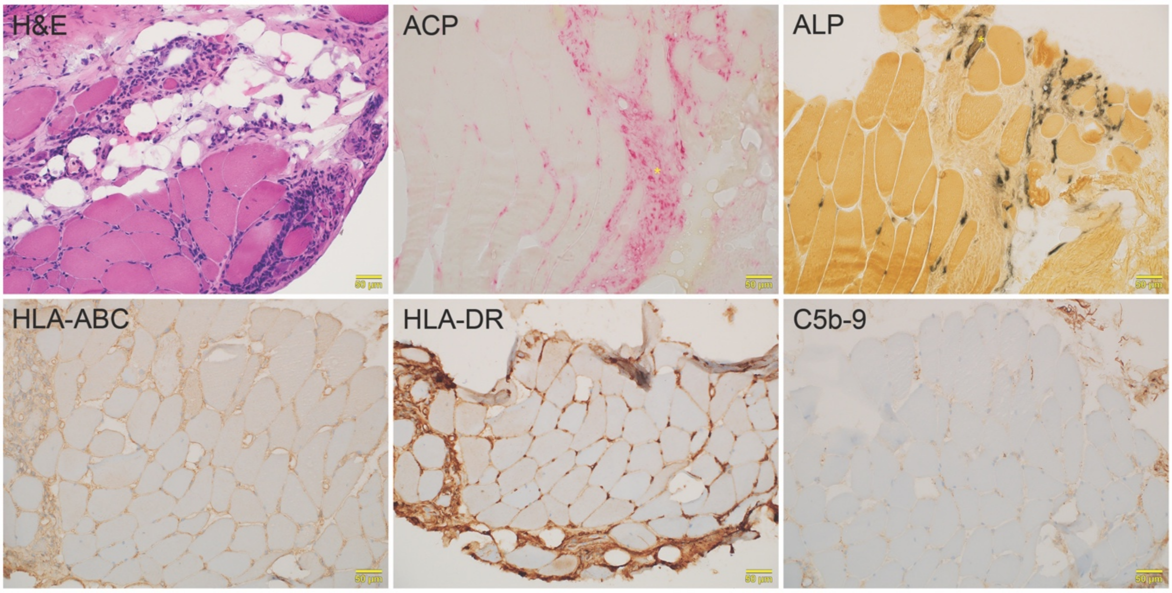

The muscle biopsy from the HLA-DR-positive FSHD patient. H&E shows fibrofatty infiltration with inflammatory cell infiltration. Many inflammatory cells show lysosomal activity which is highlighted by ACP stain(asterisk), suggestive of macrophage in nature. ALP highlights one regenerating fiber (asterisk). There is diffuse HLA-ABC and HLA-DR expression. Faint equivocal C5b-9 capillary deposition is noted.

Note: bar = 50 μm; P-MM, possible myositis mimics; FSHD, facioscapulohumeral muscular dystrophy; H&E, hematoxylin and eosin; ACP, acid phosphatase; ALP, alkaline phosphatase; HLA-ABC, human leukocyte antigen-ABC, major histocompatibility complex class I; HLA-DR, human leukocyte antigen-DR, major histocompatibility complex class II; C5b-9, membrane attack complex.

Supplementary eFigure 4

Expression levels of genes in IFN-signaling pathway in different subtypes of idiopathic inflammatory myopathy.

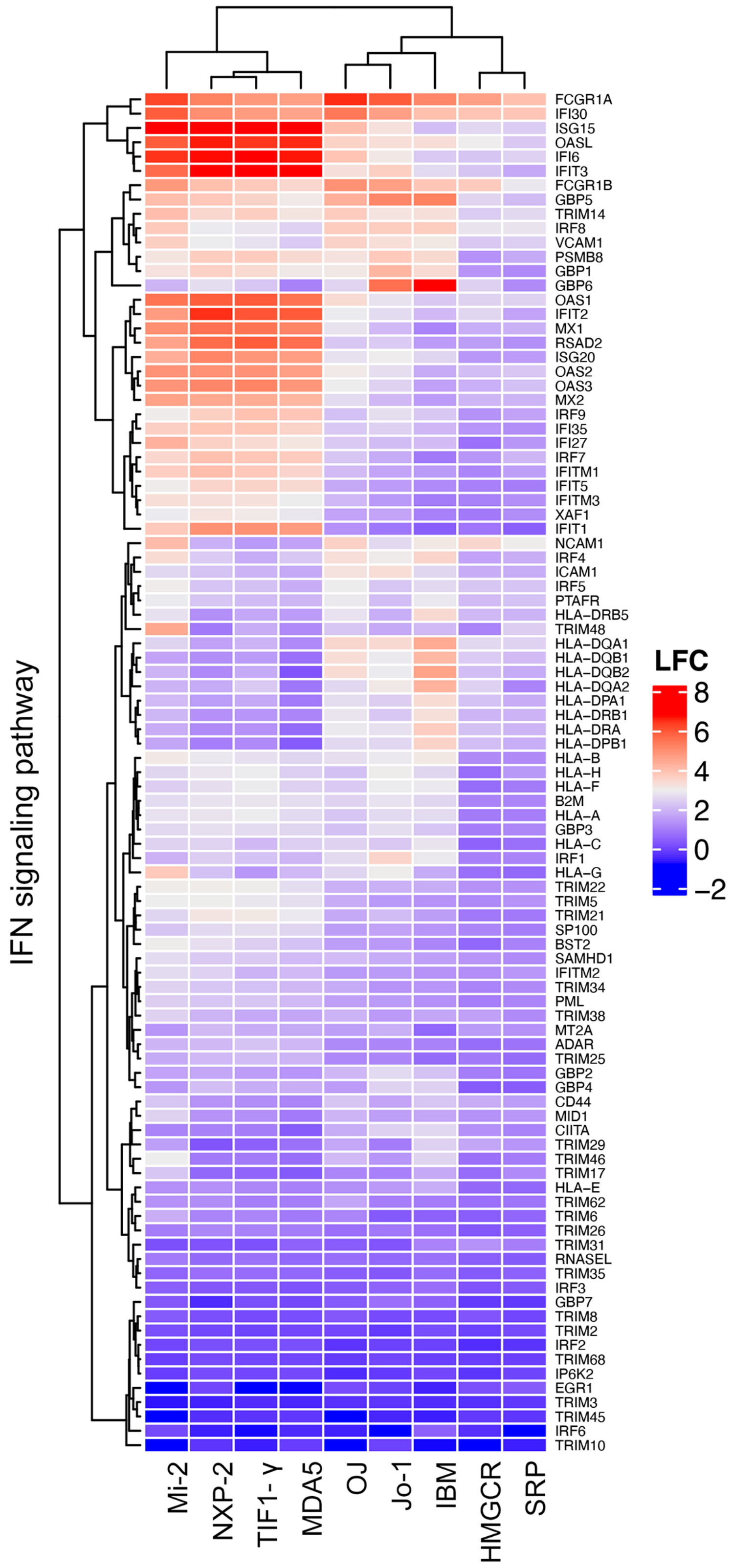

Note: IFN, interferon; Mi-2, anti-nucleosome remodeling-deacetylase (NuRD) complex; NXP-2, anti-nuclear complex protein 2; TIF1-γ, anti-transcription intermediary factor 1-gamma; MDA5, anti- melanoma differentiation-associated gene 5; OJ, anti-OJ; Jo-1, anti-Jo-1; IBM, inclusion body myositis; HMGCR, anti-3-hydroxy-3-methylglutaryl coenzyme A reductase; SRP, anti-signal recognition particle

Supplementary eFigure 5

CIITA and HLA-DR expression

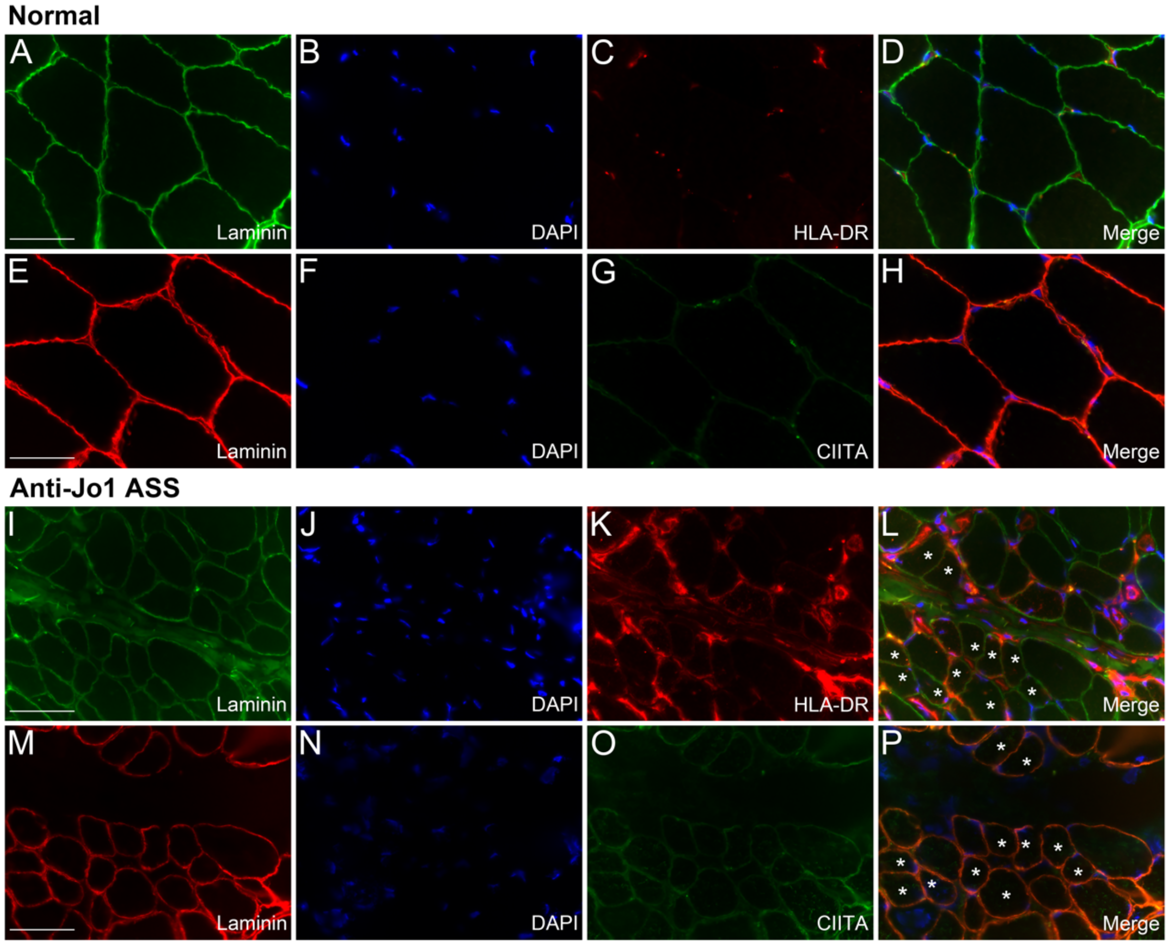

Normal muscle biopsy **(A-H):** Staining for HLA-DR and CIITA are negative in normal muscle biopsy. Serialized section of anti-Jo-1 ASS (I-P): Some fibers show HLA-DR (K) and CIITA expression (O); co-expression is focally observed (asterisks, L and P).

Note: bar = 50µm; DAPI, 4’6-diamidino-2-phenylindole; HLA-DR, human leukocyte antigen-DR, major histocompatibility complex class II; CIITA, major histocompatibility complex class II activator.
